# A DNA repair protein and histone methyltransferase interact to promote genome stability in the *Caenorhabditis elegans* germ line

**DOI:** 10.1371/journal.pgen.1007992

**Published:** 2019-02-22

**Authors:** Bing Yang, Xia Xu, Logan Russell, Matthew T. Sullenberger, Judith L. Yanowitz, Eleanor M. Maine

**Affiliations:** 1 Department of Biology, Syracuse University, Syracuse, New York, United States of America; 2 Department of Obstetrics, Gynecology, and Reproductive Sciences, Magee-Womens Research Institute, University of Pittsburgh School of Medicine, Pittsburgh, Pennsylvania, United States of America; University of Cambridge, UNITED KINGDOM

## Abstract

Histone modifications regulate gene expression and chromosomal events, yet how histone-modifying enzymes are targeted is poorly understood. Here we report that a conserved DNA repair protein, SMRC-1, associates with MET-2, the *C*. *elegans* histone methyltransferase responsible for H3K9me1 and me2 deposition. We used molecular, genetic, and biochemical methods to investigate the biological role of SMRC-1 and to explore its relationship with MET-2. SMRC-1, like its mammalian ortholog SMARCAL1, provides protection from DNA replication stress. SMRC-1 limits accumulation of DNA damage and promotes germline and embryonic viability. MET-2 and SMRC-1 localize to mitotic and meiotic germline nuclei, and SMRC-1 promotes an increase in MET-2 abundance in mitotic germline nuclei upon replication stress. In the absence of SMRC-1, germline H3K9me2 generally decreases after multiple generations at high culture temperature. Genetic data are consistent with MET-2 and SMRC-1 functioning together to limit replication stress in the germ line and in parallel to promote other germline processes. We hypothesize that loss of SMRC-1 activity causes chronic replication stress, in part because of insufficient recruitment of MET-2 to nuclei.

## Author summary

Post-translation modifications to histone proteins are known to regulate gene expression and chromosomal events such as recombination. Histone modifications are highly dynamic and are deposited by large number of histone-modifying enzymes. Little is known about how these enzymes are regulated. Using a model system, the nematode *Caenorhabditis elegans*, we show that a conserved histone-modifying enzyme, MET-2, associates with a conserved DNA repair protein, SMRC-1. In mammals, the SMRC-1 homolog, SMARCAL1, participates in repairing DNA that is damaged during replication. Focusing on the tissue responsible for production of sperm and eggs, the germ line, we find that SMRC-1 protects cells from DNA replication stress and promotes the accumulation of nuclear MET-2. Moreover, SMRC-1 affects MET-2 germline activity (as measured by histone modification state) in populations grown for multiple generations at stressful culture temperatures. Genetic analysis indicates that MET-2 and SMRC-1 participate in a common mechanism to limit DNA damage in the germ line. We propose that histone modifications are regulated to promote DNA replication and DNA repair.

## Introduction

Repetitive sequences pose challenges to genome integrity during DNA replication, DNA repair, and transcription. In eukaryotes, repetitive genomic regions typically adopt a condensed chromatin structure that is thought to limit potentially harmful consequences of repetitive sequences by limiting transcription, stabilizing DNA to promote correct repair of DNA breaks, and limiting formation of secondary structures that would otherwise impede DNA replication [1–4]. Inappropriate transcription of repetitive regions leads to DNA:RNA hybrids (R-loops), which can prevent replication fork progression. DNA break repair is particularly important at repetitive regions because homologous recombination between non-allelic repetitive sequences causes duplication/deletion of genomic regions [5, 6]. Replication of heterochromatic regions also requires modification of histones within newly incorporated nucleosomes; histone chaperones and some DNA replication factors recruit histone methyltransferases for this purpose [7, 8]. Beyond regulation at repetitive sequences, replication and chromatin state are interdependent throughout the genome, e.g., chromatin compaction influences replication fork progression [9], and conversely, impaired replication can affect chromatin modification status and reduce the accuracy of histone incorporation at sites across the genome [10, 11]. Thus, the interplay among histone modifications, DNA replication, and repetitive sequences is complex.

H3K9 methylation is a histone modification widely associated with heterochromatin [12, 13]. In *C*. *elegans*, different repetitive sequences accumulate H3K9me2 and/or H3K9me3 [14–18], and loss of these marks correlates with increased susceptibility to DNA replication stress [17]. H3K9me1 and me2 are deposited primarily by MET-2 (methyltransferase-2), the sole *C*. *elegans* member of the SETDB1 family important for heterochromatin establishment and maintenance in numerous species [19–22]. MET-2 also promotes H3K9me3 formation, perhaps indicating that H3K9me1/me2 are substrates for H3K9 trimethylation [22]. SET-25 (SET domain proteins) is responsible for H3K9me3 at other sites [21, 22], and SET-32 is required for H3K9me3 in the initiation of heritable RNA-based transcriptional silencing [23–25]. Genetic studies indicate that MET-2, alone or together with SET-25, promotes germline viability and is critical for fertility in strains maintained at elevated culture temperatures over numerous generations [17, 18, 21, 26]. Moreover, during meiosis, H3K9me2 is enriched on non-synapsed chromosomes, e.g., the male X chromosome, characteristic of a process termed meiotic silencing [27, 28]. Overall, H3K9 methylation at repetitive sequences appears to ensure long-term stability of the genome and production of viable gametes and offspring.

Known SETDB1 interactors include co-factors as well as proteins required for stable interaction with chromatin or for re-establishing H3K9 methylation following DNA replication. Members of the ATF7IP (activating transcription factor 7-interacting protein; also called mAM/MCAF1, Mbd-1 chromatin associated factor) protein family are SETDB1 co-factors in vertebrates [29, 30] and *Drosophila* [31]. *C*. *elegans* LIN-65 is a structurally related (but not orthologous) protein necessary for H3K9me2 deposition [32, 33] and for MET-2 nuclear import in the embryo [33]. *C*. *elegans* ARLE-14 promotes MET-2 association with chromatin [33] as do vertebrate KAP1 (KRAB-associated protein 1; also called TRIM28) and hnRNP K (heterogeneous nuclear ribonucleoprotein K) [20, 21, 34]. SETDB1 also associates with a member of the SWI/SNF ATPase family, BAF155/SMARCC1 [34], and inactivation of BAF155, or any of several other BAFs, impairs SETDB1 activity at retroviral elements [35, 36]. During replication, CAF-1 (chromatin-associated factor–1) and MBD1 (methyl-CpG binding domain protein) recruit SETDB1 to re-establish H3K9 methylation behind the replication fork [7, 37, 38]. Thus, numerous factors ensure H3K9 methylation in different contexts.

To better understand how MET-2 activity is targeted in the *C*. *elegans* germ line, we sought to identify MET-2-interacting proteins. Here we describe SMRC-1, the sole *C*. *elegans* ortholog of vertebrate SMARCAL1 (SWI/SNF-related, matrix associated, actin-dependent regulator of chromatin, subfamily A-like 1). SMARCAL1-related proteins comprise a distinct subfamily of SWI/SNF ATPases and are thought to protect genome integrity by promoting the repair and restart of stalled DNA replication forks [39–41]. *In vitro*, SMARCAL1 proteins bind single stranded (ss) DNA and can rewind DNA substrates, e.g., replication forks and D-loops at Holliday junctions, and RNA:DNA substrates, meaning they might act on R-loops [39, 42–44]. Studies in human cultured cells showed that telomere maintenance, an endogenous source of replication stress, requires SMARCAL1 activity [45]. The MET-2—SMRC-1 association is interesting given that MET-2 provides some protection against lethality caused by DNA replication stress [17, 18].

We demonstrate that SMRC-1 protects against DNA replication stress, limits accumulation of DNA breaks and mutations, and promotes germline and embryonic viability and development. Moreover, SMRC-1 promotes H3K9me2 deposition and an increase in nuclear MET-2 abundance under conditions of replication stress. Genetic data suggest MET-2 and SMRC-1 function in a common mechanism in the germ line to limit DNA damage caused by replication stress and in parallel to promote other germline processes. Our data suggest SET-25 does not promote SMRC-1-mediated processes and has a minimal role in limiting replication stress. Taken together, our data suggest that SMRC-1 recruits MET-2 to limit the adverse effects of replication stress.

## Results

To facilitate our study of SMRC-1, we generated loss-of-function *smrc-1* mutations using CRISPR-Cas9 genome editing. These included nonsense (*om136*), frameshift (*om138*, *ea8*), and deletion (*ea46*, *ea73*) alleles, each predicted to be severe loss-of-function (S1A Fig) [46–48] (see Methods). Each allele was outcrossed and maintained as a balanced heterozygote. We also epitope-tagged the endogenous *smrc-1* locus in order to analyze SMRC-1 protein expression (S1A Fig).

### SMRC-1 promotes fertility and embryonic viability

We evaluated the *smrc-1* phenotype in order to determine the importance of SMRC-1 during development. *smrc-1* mutants had reduced fertility, an increased frequency of male offspring, and reduced embryonic viability (Table 1). These phenotypes resulted primarily from the loss of maternal *smrc-1(+)* product and were more severe at elevated culture temperature. At 25°C, *smrc-1(om136)* M+Z- F1 hermaphrodites (the progeny of *smrc-1(+/-)* mothers) were viable and fertile, although they produced fewer embryos than did wildtype controls (S1 Table, Table 1). Some *smrc-1(om136)* M-Z- F2 individuals died as embryos, and survivors included a high proportion of males (a Him phenotype, typically due to X chromosome nondisjunction) (Table 1). Most viable *smrc-1(om136)* M-Z- F2 hermaphrodites were fertile but produced fewer embryos than the F1 generation (Tables 1 and 2). Most F3 embryos were non-viable (Table 1).

**Table 1:** Loss of *smrc-1* function causes temperature-sensitive defects.

Genotype	Temp °C	n	Avg clutch ± SEM	% Dead embryos	% Male
N2 wildtype	20	7	220.6 ± 6.7	0.6	0.1
*3xflag*::*smrc-1*	20	10	238.3 ± 8.5	1.0	0.1
*smrc-1(om136)* M+Z-	20	8	222.6 ± 8.2	9.2**	0.8
*smrc-1(om136)* M-Z-	20	10	155.5 ± 20.6*^$^	25.0**^$$^	3.8
*smrc-1(om138)* M+Z-	20	6	173 ± 6.8**	19.6**	1.4
*smrc-1(om138)* M-Z-	20	11	139.8 ± 9.5**^$^	26.5**	2.0
*smrc-1(om138) met-2(n4256)* M+Z-	20	11	159.4 ± 3.52**	21.2**	2.2
*smrc-1(om138) met-2(n4256)* M-Z-	20	12	11.2 ± 7.8**^$$^^##^	80.9**^$$^^##^	2.6
N2 wildtype	25	7	168.7 ± 4.8	3.1	0.1
*3xflag*::*smrc-1*	25	5	157.4 ± 1.6	3.8	0.1
*smrc-1(om136)* M+Z-	25	15	130 ± 3.9**	29.2**	4.5
*smrc-1(om136)* M-Z-	25	13	53.5 ± 9.0**^$$^	72.2**^$$^	5.5
*smrc-1(om138)* M+Z-	25	7	120.3 ± 6.7**	39.6**	3.5
*smrc-1(om138)* M-Z-	25	8	58.6 ± 13.8**^$$^	47.5**^$^	3.7
*smrc-1(om138) met-2(n4256)* M+Z-	25	12	124.2 ± 7.6**	54.1**^##^	1.4
*smrc-1(om138) met-2(n4256)* M-Z-	25	12^&^	1.6 ± 1.6	94.8	NA

Clutch size, embryonic lethality and male frequency were collected as described in methods. n, Number of broods assayed. Genotypes were compared by *t*-test.

* p < 0.05, and

** p < 0.01 compared to wildtype.

^$^ p < 0.05, and

^$ $^ p < 0.01 compared to M+Z- counterpart.

^#^ p < 0.05, and

^##^ p < 0.01 compared to *smrc-1(om138)* of the same generation.

^&^ Only 1/12 hermaphrodites produced embryos.

**Table 2:** *smrc-1* is synthetic sterile and lethal with *met-2*.

genotype	% sterile XX (n)	% dead embryos (n)	% protruding vulva (n)
*smrc-1(om136)*	7.5 (402)	30 (590)	0 (402)
*smrc-1(om138)*	8.3 (460)	26.8 (656)	0 (460)
*met-2(n4256)*	0 (217)	7.3 (234)	0 (217)
*set-25(tm5021)*	0 (335)	5.6 (355)	0 (335)
*smrc-1 met-2*	92 (301)	52.2 (624)	36.5 (301)
*smrc-1 set-25*	7.9 (189)	30.2 (278)	0 (189)
*met-2 set-25*	0 (108)	64.8 (307)	0 (108)

Data listed are for the M-Z- F2 generation (progeny of M+Z-) raised at 25°C.

We investigated the developmental defects underlying impaired *smrc-1* fertility by DAPI staining *smrc-1(om136)* M-Z- F2 adult hermaphrodites and evaluating their germ lines. Fertile F2 adults typically had normal germline organization, whereas sterile F2 adults had obvious germline defects such as abnormal nuclear morphology, reduced numbers of germ cells compared with wildtype, and/or failure to produce sperm, oocytes, or both gamete types (S2 Fig). In rare cases, germ cells were not at all visible in the adult gonad. When both sperm and oocytes were present, one or both gamete types were presumably fertilization-defective. A subset of sterile hermaphrodites had polyploid (endomitotic) oocytes in the oviduct, a phenotype that most commonly arises due to impaired ovulation [49, 50]. We conclude that SMRC-1 function promotes multiple aspects of germline development.

### SMRC-1 limits sensitivity to DNA replication stress

**Fig 1:**
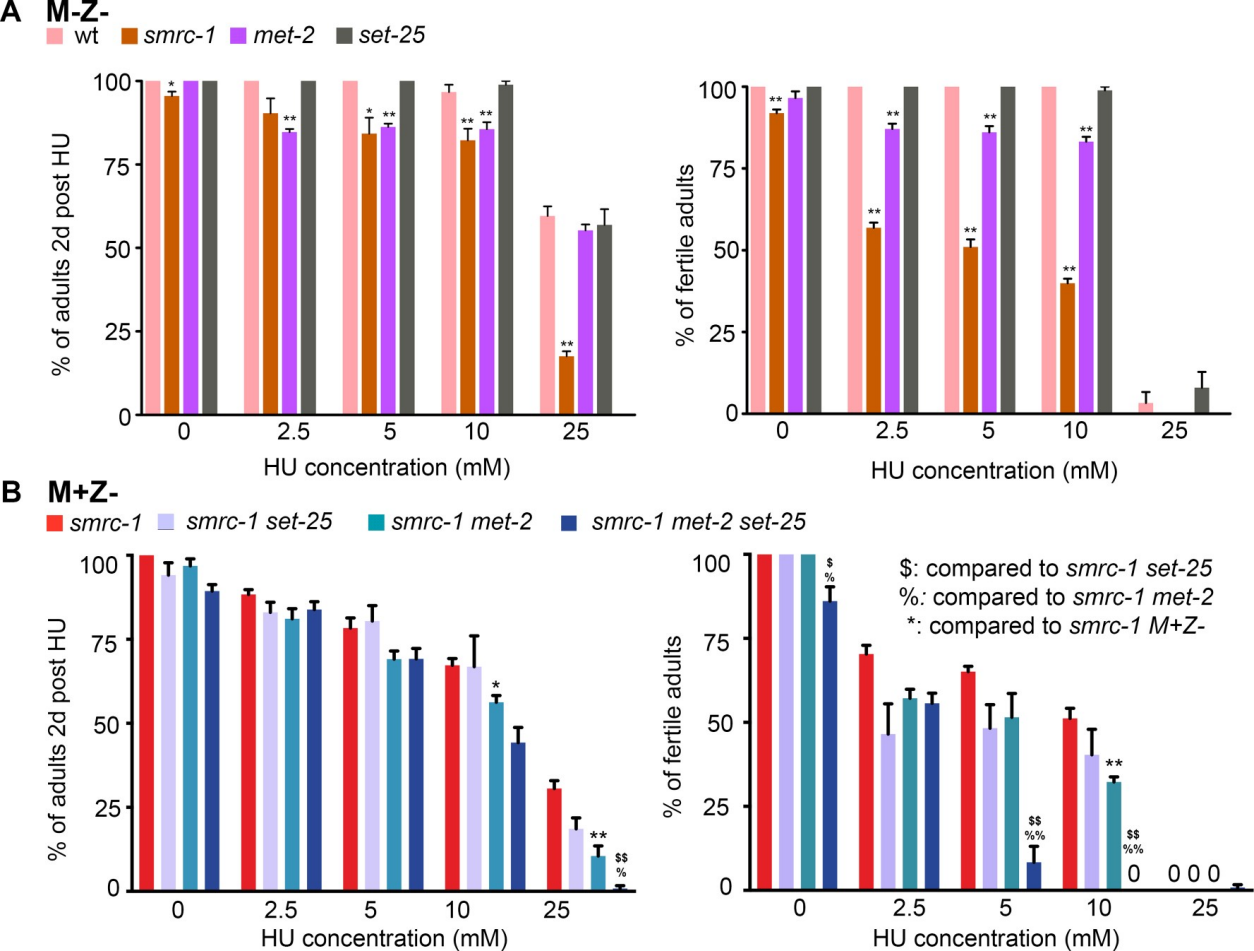
SMRC-1 reduces replication stress-induced sterility and lethality. (A) Impact of HU treatment on viability (left) and fertility (right) of *smrc-1* M-Z- F2 mutants. wt, *met-2*, and *set-25* were tested in parallel. (B) Impact of HU treatment on viability (left) and fertility (right) of M+Z- F1 generation mutants. *smrc-1* F1 M+Z- mutants, *smrc-1 set-25* M+Z- double, *smrc-1 met-2* M+Z- double, and *met-2 smrc-1 set-25* M+Z- triple mutants were tested in parallel. (A, B) Animals were exposed to HU for 16 hr at 25°C as L1 larvae, transferred to regular NGM plates, and maintained at 25°C for 48 hr. Error bars indicate standard error of the mean. *smrc-1(om136)* was used for *smrc-1* single and *smrc-1 set-25* double mutant strains; *smrc-1(om138)* was used in *smrc-1 met-2* and *smrc-1 met-2 set-25* strains. (A) Mutant and wildtype data were compared using two-tailed t-test on arcsine transformed data. * indicates *P* <0.05, ** indicates *P* <0.01. (B) Pair-wise comparison were performed as indicated in the legend. ^$^, ^%^, * indicate *P* <0.05; ^$ $^, ^%%^, ** indicate *P* <0.01. ≥ 3 biological replicates were performed with 40 L1 larvae/replicate.

We addressed the sensitivity of *smrc-1* mutants to DNA replication stress by exposing them to hydroxyurea (HU), a treatment that causes replication fork stalling. We initially examined treated *smrc-1(om136)* M-Z- F2 individuals, and later examined M+Z- F1 individuals for comparison with low-fertility genotypes. We treated larvae with HU beginning at L1 stage and monitored their survival and fertility (see Methods). Survival of L1 larvae post-HU exposure reflects their ability to resolve DNA lesions and resume development; fertility of surviving adults specifically reflects the ability of mitotic germ cells to resolve DNA lesions. HU treatment had a significantly more severe effect on viability and fertility of *smrc-1* M-Z- F2 mutants than wildtype (Fig 1A). The replication stress hypersensitivity of the *smrc-1* mutant suggests a prominent role for SMRC-1 in limiting replication-associated DNA damage.

### Transgenerational effects of SMRC-1 loss

**Fig 2:**
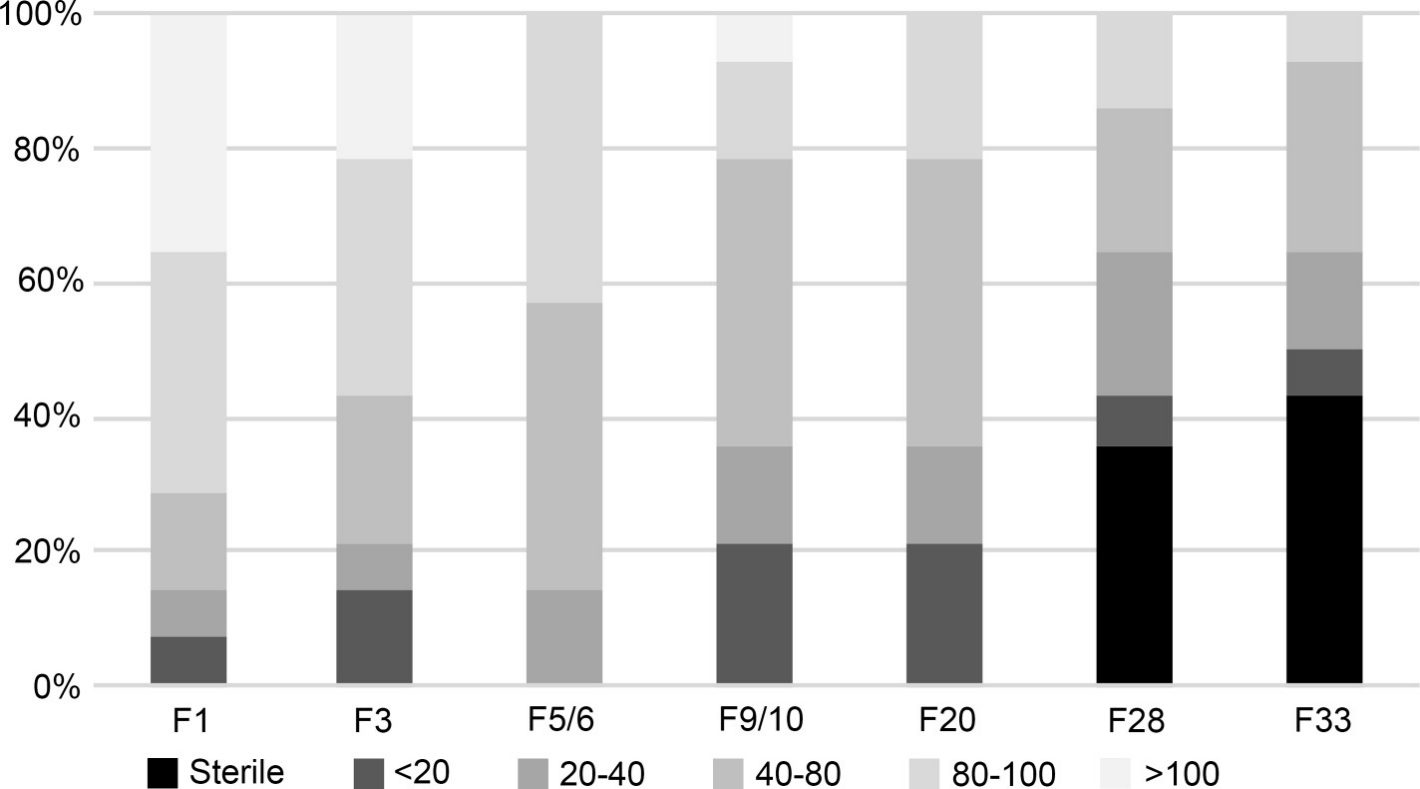
*smrc-1* mutants maintained long-term at high culture temperature have variably reduced brood sizes. 16 *smrc-1(ea8)* lines were passaged for 33 generations in parallel at 25°C. The first larva to reach L4 was passaged in each case; if sterile, a fertile sibling was chosen to rescue the line. The second generation where the oldest L4 was sterile, the line is indicated as sterile in the graph. Brood sizes were binned, as indicated. See text and Supplemental Materials and methods.

We hypothesized that *smrc-1* mutants might accumulate mutations over successive generations which would reduce survival and fertility as a result of errors due to replication stress, and possibly other sources of DNA damage [42, 51–54]. We evaluated this possibility by serially passaging 16 *smrc-1(ea8)* mutant lines at 25°C and recording brood sizes in each generation (see Methods). To eliminate bias, we passaged the first L4 larva at each generation; if that animal developed as a sterile adult, we rescued the line by passaging a fertile sibling. We observed a broad range of brood sizes at each generation among the 16 serial lines (0 to >100 offspring) (Fig 2). Eleven lines had to be rescued by siblings at least once in the course of 30 generations. Overall, there was a trend toward reduced fecundity in successive generations and populations appeared to become sicker.

### SMRC-1 limits germline apoptosis

**Fig 3:**
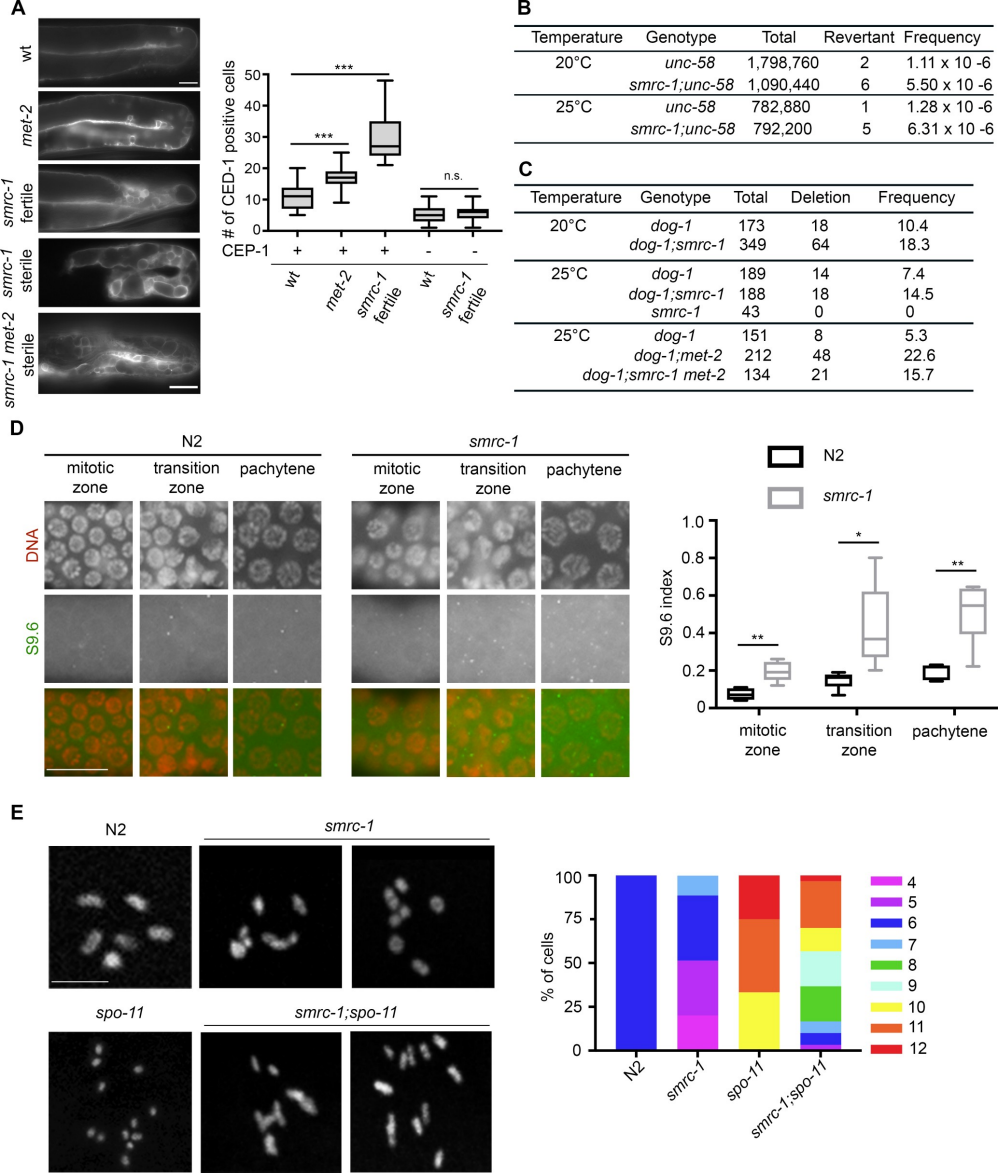
SMRC-1 promotes genome integrity. (A) Apoptotic germ cells are identified by CED-1::GFP expression in control and representative fertile and sterile *smrc-1;ced-1*::*gfp* adult gonads. Each gonad is oriented with the loop region to the right. Plots show the number of CED-1::GFP positive cells per gonad arm in wildtype, *met-2*, and fertile *smrc-1* mutants; box represents the middle 50% of values, line represents the 50^th^ percentile (median) value, and bars indicate the full range of values. CED-1::GFP is observed throughout sterile *smrc-1* and *smrc-1 met-2* gonads; these individuals were not quantified because it was difficult to reliably distinguish individual cells. Elevated apoptosis is CEP-1-dependent. ***P<0.001, Student’s t-test. N.s., not significant. See S2C Fig for more *smrc-1 met-2* images. (B) Reversion of the *unc-58(e665)* phenotype is enhanced by *smrc-1* loss-of-function at 20° and 25°C. Total = number of individuals assayed. (C) The *dog-1* poly G/C deletion phenotype is elevated in *smrc-1* and *met-2* mutants. Data represent the deletion frequency within *vab-1* exon 5. Total = number of individuals assayed. Deletion = number of deletions identified. Note that *smrc-1 met-2* double mutants were assayed in the M+Z- generation. (D) Antibody S9.6 immunolabeling detects elevated RNA:DNA hybrids throughout the *smrc-1* germ line. The proportion of nuclei with S9.6 foci (the S9.6 index) was calculated independently for proliferative, leptotene/ zygotene (transition), and pachytene regions of 25°C wildtype and *smrc-1* M-Z- hermaphrodite germ lines. *P<0.02, **P<0.002. (E) *smrc-1* mutations alter bivalent formation. Images show representative diakinesis-stage nuclei in the -1 oocyte. Wildtype image contains 6 larger DAPI-bright bodies, representing 6 bivalents, and *spo-11(ok79)* image contains 12 smaller DAPI-bright bodies, representing 12 univalents. Righthand panel shows the number of DAPI-stained bodies per diakinesis nucleus in strains raised at 25°C. *smrc-1(om138)* and *smrc-1(ea8)* oocytes contain 4–7 distinct DAPI-bright bodies of variable size; larger bodies often appear to contain chromosomes held together by DNA bridging (arrows). DNA linkages were verified by rotation of confocal microscopy Z-stacks. *smrc-1(om138);spo-11(ok79)* oocytes contain 5–12 DAPI-bright bodies of variable size.

Germ cell apoptosis is elevated in many *C*. *elegans* DNA damage response mutants [55], and thus we considered that elevated apoptosis might contribute to the reduced *smrc-1* fertility. We evaluated apoptosis by monitoring expression of CED-1::GFP, a protein expressed on the surface of phagocytic cells as they engulf apoptotic cells [56], and by staining with the vital dye, acridine orange. Here, CED-1 is expressed by sheath cells, components of the somatic gonad that engulf apoptotic germ cells. Both assays revealed elevated levels of apoptosis in *smrc-1* germ lines compared to controls (Fig 3A, S2 Table). In the *C*. *elegans* hermaphrodite gonad, CED-1 is expressed by somatic sheath cells adjacent to the germ line [56]. As expected based on the literature, we observed rare CED-1::GFP -positive cells at the loop region of wildtype gonad (Fig 3A). *smrc-1* mutants contained significantly more CED-1-positive cells, often present throughout the germ line (Fig 3A). *smrc-1* apoptosis was significantly reduced in the absence of CEP-1/p53 (Fig 3A, S2 Table), an essential component of the DNA damage checkpoint machinery active at the late pachytene stage [57]. In contrast, apoptosis was not significantly suppressed by inactivation of PCH-2 (S2 Table), a component of the machinery that monitors chromosome pairing [58]. We conclude that *smrc-1* mutants accumulate unrepaired DNA damage that, in turn, triggers the DNA damage checkpoint and results in elevated germline apoptosis.

### SMRC-1 limits the accumulation of mutations

*C*. *elegans* mutations that cause DNA damage to accumulate, due to either an increased number of DNA lesions or impaired DNA damage repair machinery, are classified as “mutators” [59]. We hypothesized that SMRC-1 might limit accumulation of mutations. We investigated this possibility in two ways. First, we screened for reversion of the dominant *unc-58(e665)* phenotype; this assay allows detection of intragenic and extragenic suppressors and is commonly used to quantify mutator activity [60]. We observed a 3- to 5-fold increase in *unc-58* reversion in the *smrc-1* mutant background compared to wildtype at both 20°C and 25°C (Fig 3B). For comparison, reversion increased 8- to 15-fold in DNA damage response mutants *clk-2*, *hus-1*, and *mrt-2* [60]. Second, we assayed for enhancement of the *dog-1* phenotype. DOG-1 (deletions of G-rich DNA) helicase is related to human FANCJ and essential for proper replication of poly G/C tracts in *C*. *elegans* [61]. Poly G/C tracts can assume a DNA secondary structure that is a natural source of replication stress [62]. Proteins that function in the DNA damage checkpoint or homologous recombination, e.g., CEP-1 or XPF-1 and SWS-1, respectively, are implicated in maintaining poly G/C tract integrity in the absence of *dog-1* activity [63–65]. We evaluated poly G/C tract integrity by assessing the deletion rate within G/C-rich exon 5 of the *vab-1* gene. *vab-1* microsatellite deletions or insertions did not accumulate in *smrc-1(ea8)* or *smrc-1(ea46)* single mutants (Fig 3C). In contrast, deletion frequency was two-fold higher in *dog-1;smrc-1* double mutants compared to *dog-1* single mutants at both 20°C and 25°C (Fig 3C). For comparison, deletion frequency was increased 2.7-fold in *cep-1*, 3.2-fold in *xpf/him-9*, and 1.5-fold in *cep-1* mutants compared to wildtype [63, 65]. We conclude that SMRC-1 limits the accumulation of deletions within poly G/C regions when DOG-1 activity is absent.

### SMRC-1 limits R-loop accumulation

*In vitro* studies suggest that mammalian and *Drosophila* SMARCAL1 may act on DNA:RNA hybrids (43), and R-loops cause DNA replication stress *in vivo* (5). We were interested in determining if SMRC-1 might limit accumulation of R-loops. We evaluated R-loop abundance in wildtype and *smrc-1(om138)* M-Z- F2 mutants by immunolabeling with a DNA:RNA hybrid-specific antibody, S9.6 [66]. We quantified the proportion of nuclei with S9.6 foci (the S9.6 labeling index) in the proliferative, leptotene/zygotene, and pachytene regions of the germ line. *smrc-1* germ lines had a significantly greater S9.6 labeling index than wild type in each of these regions (Fig 3D). Moreover, the S9.6 -positive *smrc-1* germ line nuclei had more S9.6 foci on average than the positive wildtype nuclei (Fig 3D). We interpret these data to indicate that SMRC-1 activity limits R-loop abundance.

### SMRC-1 limits formation of SPO-11-independent double-strand breaks

We considered whether the loss of SMRC-1 function in proliferating germ cells might contribute to formation of DSBs that could impact genome integrity. In early *C*. *elegans* meiosis, SPO-11 endonuclease initiates DSB formation at multiple sites along each chromosome; in most nuclei, only one DSB per chromosome is repaired as a crossover (CO) and others are repaired as noncrossovers (NCOs) [67, 68]. COs do not occur and homologs prematurely dissociate at diakinesis if SPO-11 is absent [67, 68]. Introduction of DSBs from exogenous sources, such as ionizing radiation (IR), can partially rescue COs in *spo-11* mutants [67, 68].

We took advantage of the *spo-11* univalent phenotype to test whether unregulated (non-SPO-11-mediated) DSBs might arise in *smrc-1* mutants and contribute to the loss of germline viability. First, we generated *smrc-1; spo-11* double mutants and evaluated diakinesis chromosomes in the most proximal oocyte (at the -1 position) in each gonad arm. In *smrc-1(ea8)* and *smrc-1(om138)* single mutants, we observed 4–7 DAPI-bright bodies in the -1 oocyte (Fig 3E). Faint links occasionally were visible between what appeared to be distinct chromosomes in *smrc-1* mutant oocytes, suggesting aberrant connections between non-homologous chromosomes (Fig 3E). The presence of 7 DAPI-bright bodies in some nuclei is consistent with five synapsed autosomal pairs and two non-synapsed X chromosomes, which would lead to nullo-X gametes and subsequent production of male offspring, as observed. In *smrc-1(om138);spo-11(ok79)* double mutants raised at 25°C, we observed striking evidence of additional DNA damage. We observed 5–12 DAPI-bright bodies, and more frequent faint links between chromosomes (at least one linkage observed in 22% of nuclei, N = 41) (Fig 3E). Therefore, SMRC-1 appears to limit production of aberrant DNA damage that could be carried into meiosis and allow inappropriate connections between chromosomes.

RAD-51 is a single-strand DNA binding protein that associates with ssDNA adjacent to DSBs and facilitates the homology search and strand engagement in homologous recombination [69]. We performed anti-RAD-51 labeling to evaluate the distribution of DSBs in the *smrc-1(om138)* M-Z- F2 germ line. We observed RAD-51 foci primarily in meiotic nuclei and more rarely in mitotic nuclei, similar to wild-type controls (S4A Fig). Several differences were noted, however. Specifically, while DSBs occurred in leptotene stage in both wild type and *smrc-1* mutants, RAD-51 foci formation was delayed in *smrc-1*. One possibility is that SPO-11-induced breaks occurred over a more protracted period of time in *smrc-1* mutants. Also, a large number of RAD-51 foci persisted into late pachytene (zone 6) in *smrc-1*, suggesting that DSB repair is delayed, as might be expected for the non-SPO-11 mediated DNA damage (described above). Finally, the total number of RAD-51 foci was elevated in *smrc-1* mutants compared with wild type. This increase is consistent with the presence of both SPO-11-mediated and pre-meiotic DNA damage-associated DSBs in the *smrc-1* germ line.

We next evaluated RAD-51 foci in *smrc-1;spo-11* meiotic nuclei as a means of visualizing aberrant DSBs. Few RAD-51 foci were observed in the *spo-11(ok79)* negative control, as expected [70]. RAD-51 foci were substantially more abundant in *smrc-1(om138);spo-11(ok79)* and *smrc-1(om136);spo-11(me44)* germ lines, particularly in leptotene-pachytene nuclei (S3A Fig). These foci may represent DSBs that formed due to DNA damage during mitosis or pre-meiotic S phase. We note that RAD-51 foci were more abundant at late pachytene nuclei in *smrc-1* single mutants, which presumably contain both SPO-11-induced and aberrant DSBs. This result raises the possibility that *smrc-1* is required for the normal processing/repair of meiotic DSBs.

### SMRC-1 affects meiotic recombination

The presence of elevated RAD-51 foci prompted us to ask whether meiotic CO events might have an altered distribution in *smrc-1* mutants. For *C*. *elegans*, CO frequency is significantly greater on the autosomal arms than in the chromosome centers [71]. We first assayed CO frequency in control and *smrc-1* animals in two small intervals within the central region of chromosome I. Our data indicated a several-fold increase in recombination between visible marker mutations in these intervals in *smrc-1* mutants compared to controls (S3B Fig). We next mapped CO distribution along the length of chromosome I by assaying single nucleotide polymorphisms (SNPs). We generated a *smrc-1* allele in the polymorphic CB4256 strain background and evaluated SNPs distributed across chromosome I (S1A Fig; see Materials and methods). This strategy allowed us to measure recombination within five large intervals along the chromosome (S3C and S3D Fig). The recombination frequency in these large intervals was not statistically different from controls except for a significant decrease in recombination frequency within interval 4 (P<0.03). Strikingly, we observed a >7-fold increase in the frequency of double CO events in the *smrc-1* background relative to the control, which indicates impaired CO homeostasis. The differences obtained with the two mapping strategies could be explained by the size of the regions assayed; the domains with the genetic markers are both contained within the -8.5cM– 5cM region assayed by the chromosome-wide analysis. By interrogating a large domain with out cytological assay, fluctuations in recombination at the local level may be buffered by compensatory changes in nearby regions. Alternatively, the locally elevated CO rates between the visible markers might be explained by the presence of sequences that are prone to breakage, e.g., microsatellite repeats, are located between the pairs of visible markers. Indeed, numerous microsatellite repeat sequences are located between *unc-11* and *dpy-5*, including a ~14.8 kb cluster (at chromosomal position 4280037–4294876); several smaller microsatellite repeat clusters are located between *dpy-5* and *unc-13* (www.wormbase.org). Such sequences could also account for the increased frequency of double COs.

### SMRC-1 associates with MET-2

**Fig 4:**
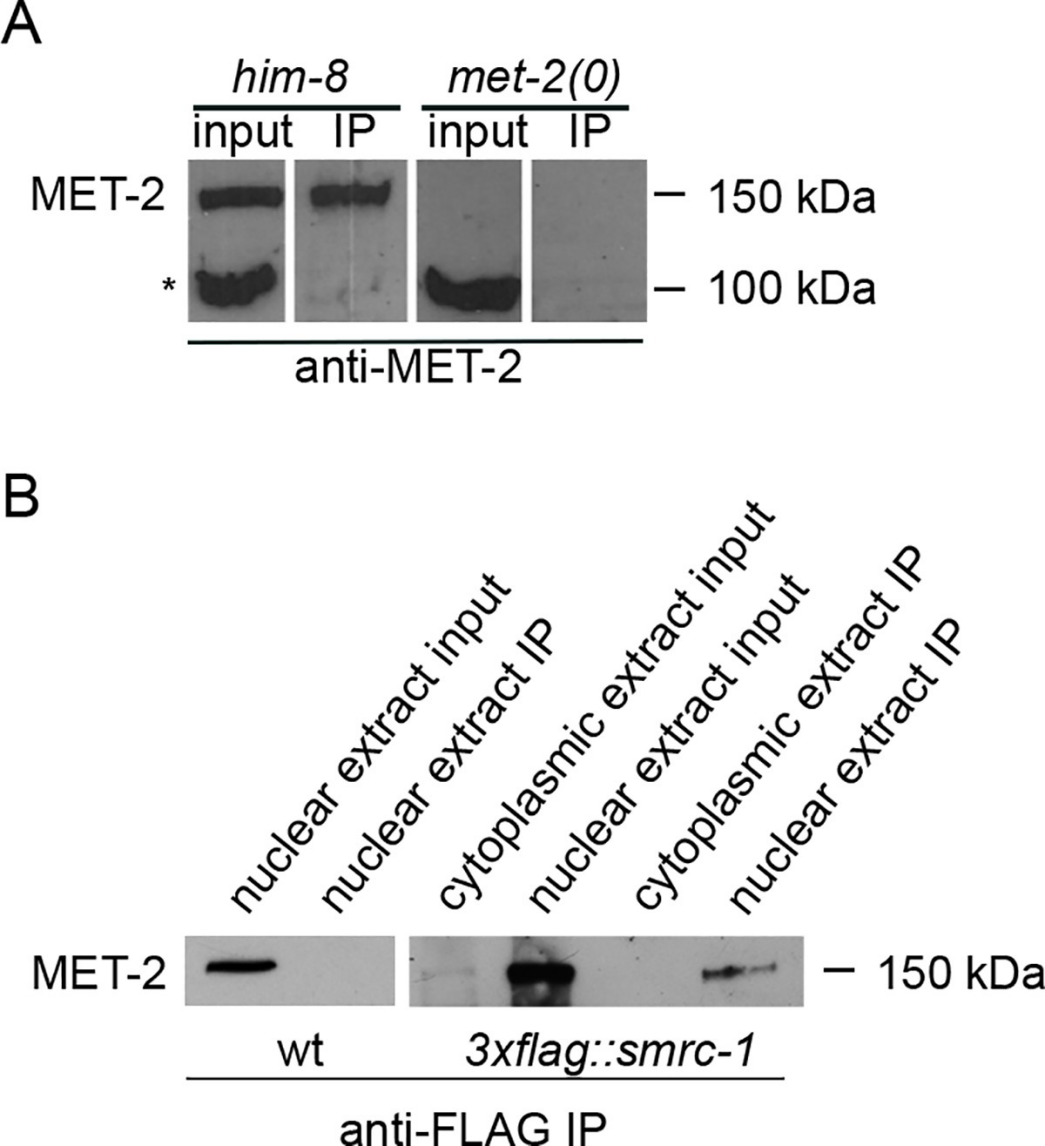
SMRC-1 associates with MET-2. (A) Protein blot containing total lysate (left two lanes) and immunoprecipitated material (right two lanes) was probed with anti-MET-2 antibody. Nuclear protein extracts were prepared from *him-8(e1489)* (control) and *met-2(n4256)* mutants, and IP was performed with anti-MET-2 antibody. *, Cross-reacting polypeptide routinely observed on protein blots but not recovered in IP under our conditions. Images are from a single immunoblot with uninformative lanes cropped out. (B) Protein blots containing total nuclear and cytoplasmic lysates and material immunoprecipitated with anti-FLAG antibody were probed with anti-MET-2. Extracts were prepared from wildtype and *3xflag*::*smrc-1* adults, as indicated.

Our attention was originally drawn to SMRC-1 as a consequence of our co-immunoprecipitation (co-IP) studies designed to identify MET-2-associated proteins. In these studies, we performed IPs using anti-MET-2 polyclonal antibody (described in Mutlu *et al*., 2018) and consistently recovered a protein of the expected size, ~150 kD, that was absent from *met-2(n4256)* negative controls (Fig 4A). SMRC-1 was recovered in these assays. To validate the association, we *3xflag*-tagged the endogenous *smrc-1* gene using CRISPR-Cas9 genome editing (S1A Fig) and performed anti-FLAG co-IP. We consistently recovered MET-2 in the 3xFLAG::SMRC-1 co-IP (Fig 4B).

### SMRC-1 and MET-2 localize to mitotic and meiotic germ cell nuclei

**Fig 5:**
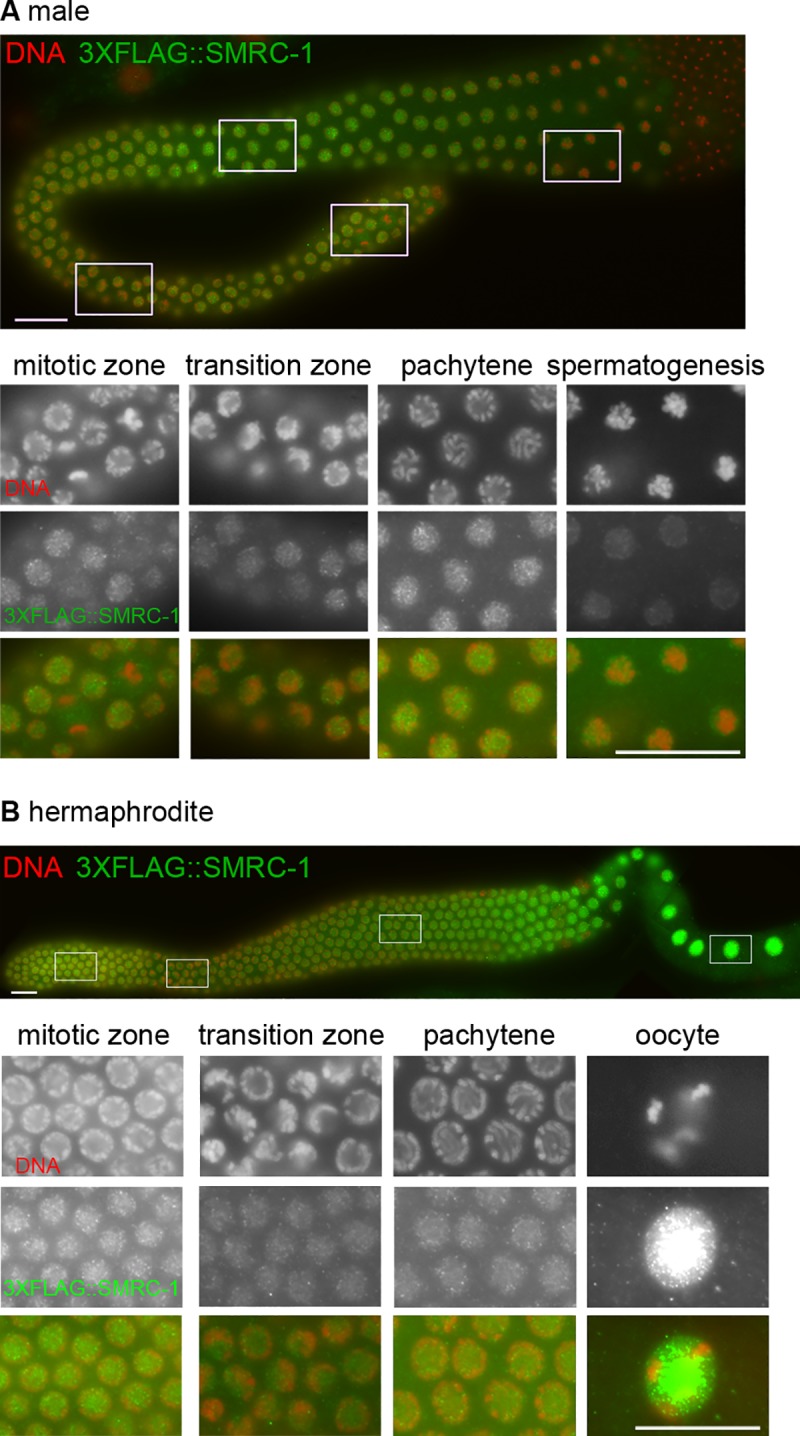
SMRC-1 localizes to germ cell nuclei. Images show dissected adult gonads labeled with anti-FLAG antibody (green), counterstained with DAPI (red), and visualized with epifluorescence microscopy. (A) A complete XO male gonad is shown above; lower panels correspond to the boxed images. (B) An XX hermaphrodite gonad is shown above; lower panels correspond to the boxed regions. Nuclear 3xFLAG::SMRC-1 is detected throughout the (A) male and (B) hermaphrodite germ lines. Scale bars: 16 μm.

We investigated SMRC-1 and MET-2 distribution in the germ line to identify where they are co-expressed. We visualized SMRC-1 by immunolabeling dissected *3xflag*::*smrc-1* gonads. We note that *3xflag*::*smrc-1* animals developed normally and had brood sizes similar to controls, suggesting the epitope tag did not substantially impact SMRC-1 function (Table 1). We detected 3xFLAG::SMRC-1 in proliferative and meiotic germ cell nuclei in XO males and XX hermaphrodites (Fig 5A and 5B). In males and hermaphrodites, labeling intensity decreased as nuclei transitioned from the proliferative region into early meiosis (leptotene-zygotene stages) and then increased again as nuclei moved through pachytene and diplotene stages. In males, signal decreased again during the condensation phase of spermatogenesis and moved to the nuclear periphery, well apart from chromatin (Fig 5A). In hermaphrodites, signal intensity was strongest in diakinesis stage oocytes, consistent with embryos inheriting substantial SMRC-1 protein (Fig 5B).

**Fig 6:**
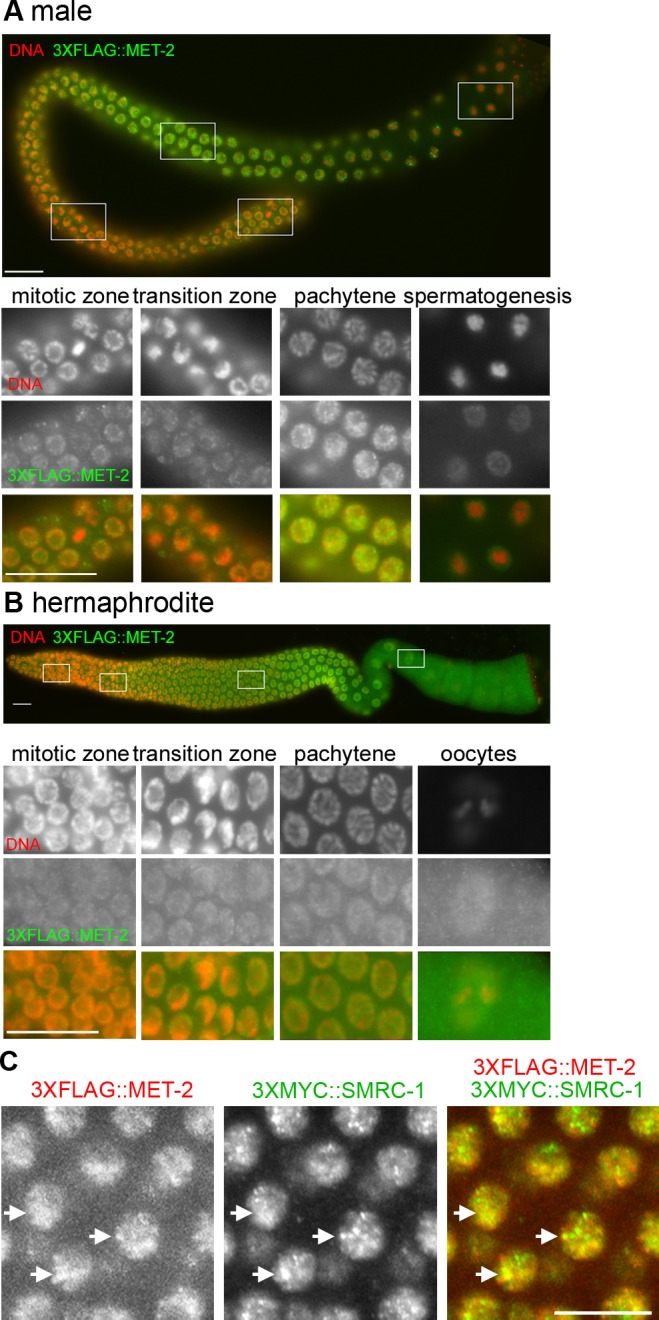
Nuclear MET-2 is detected throughout the XO and XX germ line. Images show dissected adult gonads labeled with anti-FLAG antibody (green) and counterstained with DAPI (red). (A) A complete male gonad is shown above; lower panels correspond to the boxed images presented in distal to proximal order. (B) A hermaphrodite gonad is shown above; lower panels correspond to the boxed regions. Scale bar: 16 μm. (C) Pachytene germ cells co-labeled for 3xMYC::MET-2 and 3xFLAG::SMRC-1. Images show a portion of a dissected adult germ line immunolabeled with anti-MYC and anti-FLAG antibodies, counterstained with DAPI, and visualized with confocal microscopy. Single-label images are shown in grey scale. Merged image: MET-2 (red), SMRC-1 (green). Scale bar: 5 μm. Arrows indicate example of regions with co-labeling. See S4 Fig for additional images.

We visualized MET-2 with several reagents, including anti-MET-2 antibody, epitope-tagged transgene generated by *mosI*-mediated single copy insertion (mosSCI), and endogenously-tagged MET-2 generated by CRISPR-Cas9 editing (S1A Fig; Materials and methods) [33] These reagents detected nuclear MET-2 throughout the germ line (Fig 6, S1B–S1D Fig), consistent with both our previous observation of nuclear MET-2 in embryonic nuclei using the same reagents [33] and also examination of adult somatic tissue [72]. We observed MET-2 puncta superimposed on a more diffuse signal in germline nuclei and, to a lesser extent, cytoplasm in both male (Fig 6A) and hermaphrodite (Fig 6B) germ lines. The MET-2 distribution appeared to shift as germ cells moved from the proliferative region into and through meiosis; nuclear puncta were more obvious in mitotic and leptotene-zygotene nuclei, and the signal became more evenly distributed as nuclei entered and progressed through pachytene stage (Fig 6). We conclude that germline MET-2 comprises nuclear and cytoplasmic pools. Nuclear puncta resemble the nuclear hubs observed in embryos which are thought to be sites of methyltransferase activity [33].

We co-visualized SMRC-1 and MET-2 using a *3xmyc*::*smrc-1 3xflag*::*met-2* strain generated by CRISPR-Cas9 genome editing (S1A Fig). Labeling this strain verified that SMRC-1 and MET-2 are expressed in the same nuclei (Fig 6C, S4 Fig). We observed partial overlap between 3xMYC::SMRC-1 and 3xFLAG::MET-2 signals within nuclei as would be expected for proteins that physically associate (Fig 6C, S4 Fig).

### DNA replication stress increases SMRC-1 and MET-2 abundance in germline proliferative zone nuclei

**Fig 7:**
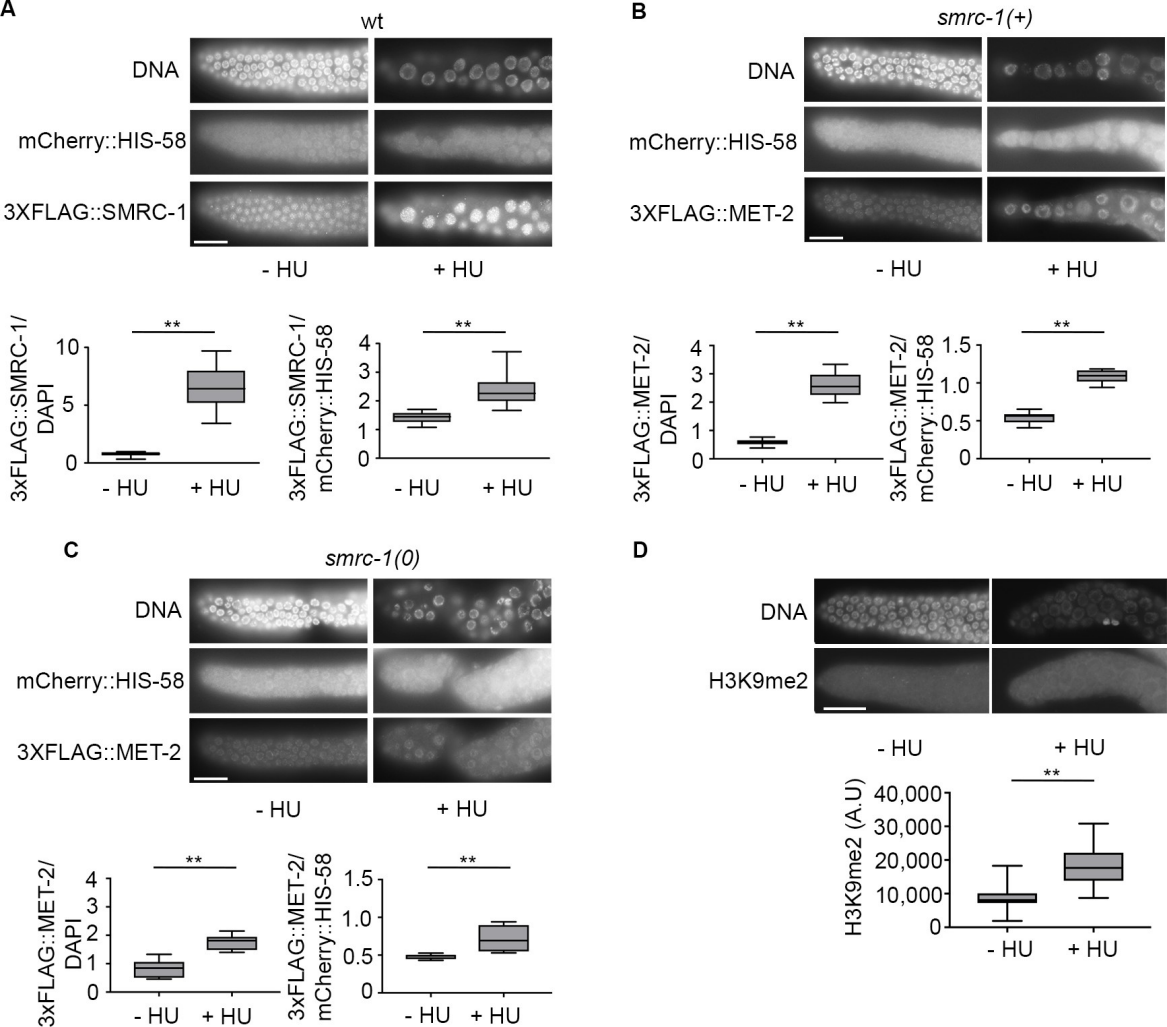
Nuclear SMRC-1 and MET-2 abundance in distal germline nuclei is elevated upon hydroxyurea treatment. (A) 3xFLAG::SMRC-1 abundance in distal germline nuclei with/without HU treatment. Images show anti-FLAG immunolabeled gonads from control and HU-treated adults. Gonad arms are oriented with the distal end to the left (A-D). L4 larvae were exposed to 25mM HU for 24 hours at room temperature (22°C) prior to immunolabeling. Box and whisker plots show normalized 3xFLAG::SMRC-1 signals with/without HU treatment; box represents the middle 50% of values, line represents the 50^th^ percentile (median) value, and bars indicate the full range of values. SMRC-1 signal is shown normalized to mean DAPI fluorescence intensity (left) and mCherry::HIS-58 fluorescence intensity (right). For each germ line, 5–7 mitotic nuclei in a similar state of chromatin condensation and a common focal plane were measured; nuclei were random with respect to size. 6–8 germ lines were measured per treatment and per replicate (see Supplemental materials and methods). See S5 Fig for normalization to total histone H3. N = 3 biological replicates. (B, C) 3xFLAG::MET-2 abundance in distal germline nuclei with/without HU treatment in (B) control and (C) *smrc-1* mutant germ lines. HU treatment and quantification were performed as in (A). N = 2 biological replicates. (D) H3K9me2 abundance in distal nuclei is higher following HU treatment. **, P<0.01.

Human SMARCAL1 localizes to stalled replication forks and forms foci in response to HU treatment of cultured cells [41]. We tested the impact of stalled replication on SMRC-1 distribution by comparing the 3xFLAG::SMRC-1 signal in germ cells with and without HU treatment. For this assay, we treated L4 larvae with 25mM HU for 24 hours at 22°C and then dissected and immunolabeled the adult gonads. Germ cell nuclei located distal to the leptotene/zygotene region were enlarged and appeared to have ceased mitosis, consistent with robust activation of the replication checkpoint (Fig 7A). We quantified the anti-FLAG signal and normalized it relative to (i) DAPI, (ii) mCherry-tagged histone H2B included in the strain background, and (iii) histone H3. We consistently observed elevated SMRC-1 abundance in distal nuclei of HU-treated animals (Fig 7A, S5 Fig), suggesting that DNA replication stress triggered an increased SMRC-1 abundance during mitosis.

Zeller et al. (2016) reported that *met-2 set-25* double mutants have reduced viability following HU treatment, suggesting that H3K9 methylation offers protection from replication stress [17]. DNA damage has been shown in other systems to increase H3K9me2 levels in other systems [3]. Regulated nuclear import of MET-2 is one way in which its activity is controlled in the embryo [33]. Given these observations, we hypothesized that nuclear MET-2 abundance in the proliferative germ line might increase under conditions of replication stress. We tested this idea by treating *3xflag*::*met-2* L4 larva with HU (as described above for *3xflag*::*smrc-1*, see Methods) and visualizing 3xFLAG::MET-2 by immunolabeling. We reproducibly observed elevated MET-2 levels in mitotic germ cell nuclei of HU-treated animals compared to untreated controls (Fig 7B). We conclude that replication stress triggers an increased MET-2 accumulation in nuclei of proliferative germ cells.

We performed anti-H3K9me2 labeling to determine if the increase in nuclear MET-2 correlates with increased activity. As previously reported, we observed weak or no H3K9me2 signal in the proliferative germ line and any signal that was present tended to be punctate and located near the nuclear periphery ([21, 27, 73]; this study). In HU-treated germ lines, we observed weak, diffuse labeling that tended to be located more centrally (Fig 7D). To compare the H3K9me2 signal in these two sets of nuclei, we modified the Corrected Total Cell Fluorescence calculation previously developed to compare cellular immunolabeling signal to calculate specifically a Corrected Total Nuclear Fluorescence (CTNF) value (Fig 7D) (see Materials and methods). The CTNF was significantly greater for distal nuclei that had received the HU treatment, suggesting that replication stress led to an increase in H3K9me2 levels.

### SMRC-1 promotes an increase in MET-2 abundance upon replication stress

Since nuclear SMRC-1 and MET-2 levels increase in the distal germline upon replication stress, we asked if SMRC-1 promotes the MET-2 increase. For this purpose, we generated a strain carrying the *smrc-1(om138)* mutation in a *3xflag*::*met-2* background and assayed the impact of HU treatment. We observed a significant increase in nuclear MET-2 abundance, however the increase was less pronounced and more variable than in *smrc-1(+)* controls (Fig 7C). These results are consistent with SMRC-1-dependent and -independent regulation of nuclear MET-2 accumulation during replication stress.

**Fig 8:**
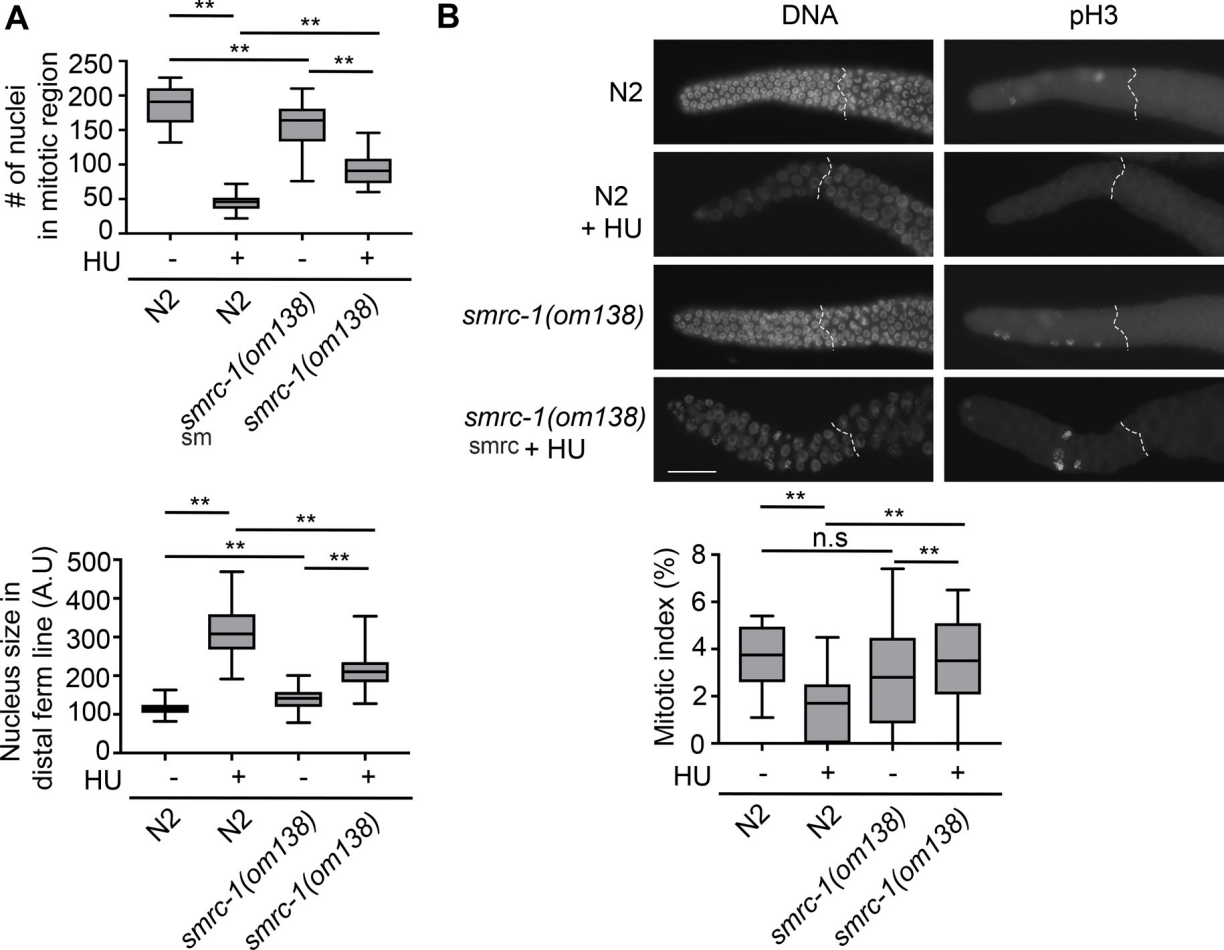
*smrc-1* germ cells are resistant to mitotic arrest. (A) In wildtype and *smrc-1* mutants, HU treatment significantly reduced the number of distal germ cell nuclei and increased their size. However, the response in *smrc-1* was significantly milder than in wt germ cells. Plots show number of nuclei in the mitotic region (above) and average nuclear area (below); box represents the middle 50% of values, line represents the 50^th^ percentile (median) value, and bars indicate the full range of values. (B) wt and *smrc-1* have the same mitotic index under standard growth conditions. Mitotic germ cells were identified by anti-H3S10phos labeling (pH3). Plot shows the mitotic index (number of H3S10phos-positive nuclei distal to meiotic entry/ total number of nuclei distal to meiotic entry); box represents the middle 50% of values, line represents the 50^th^ percentile (median) value, and bars indicate the full range of values. Upon HU treatment, there is a significant reduction in the mitotic index in wildtype but not in *smrc-1*. **, Mitotic index of HU-treated wt and *smrc-1* germ cells is significantly different, *P*<0.05. N = 14–20 germ lines assayed per treatment.

In the course of these experiments, we noted that *smrc-1* and wildtype germ cells responded differently to HU treatment. In wildtype, distal germline nuclei became notably enlarged and decreased in number, as reported in the literature (e.g., [74]). The size increase and number reduction were less pronounced in *smrc-1* nuclei, although still significant (Fig 8A). To investigate if *smrc-1* mutants were resistant to mitotic arrest, we repeated the HU treatment and performed anti-H3S10phos (histone H3 phosphorylated on serine 10) labeling to detect mitotic nuclei [75]. Untreated wildtype and *smrc-1(om138)* mutants had the same mitotic index, whereas HU-treated *smrc-1* mutants had a significantly higher mitotic index than HU-treated wildtype controls (Fig 8B). Hence, *smrc-1* germ cells appear to be resistant to mitotic arrest. We note that the failure to elicit a cell cycle arrest is not due to an inability to respond to HU, as there was a decrease in mitotic nuclei numbers of HU exposure in *smrc-1* mutants. Failure of mitotic arrest may explain why MET-2 abundance does not increase as much in the distal germ line of HU-treated *smrc-1* mutants as it does in HU-treated wildtype. Mitotic arrest occurs when the mitotic DNA damage checkpoint has been tripped; this checkpoint is HUS-1-dependent and distinct from the later DNA damage checkpoint that triggers apoptosis [76]. Perhaps SMRC-1 promotes the mitotic DNA damage checkpoint, and hence the resistance to mitotic arrest observed in smrc-1 mutants.

### SMRC-1 and germline H3K9me2

**Fig 9:**
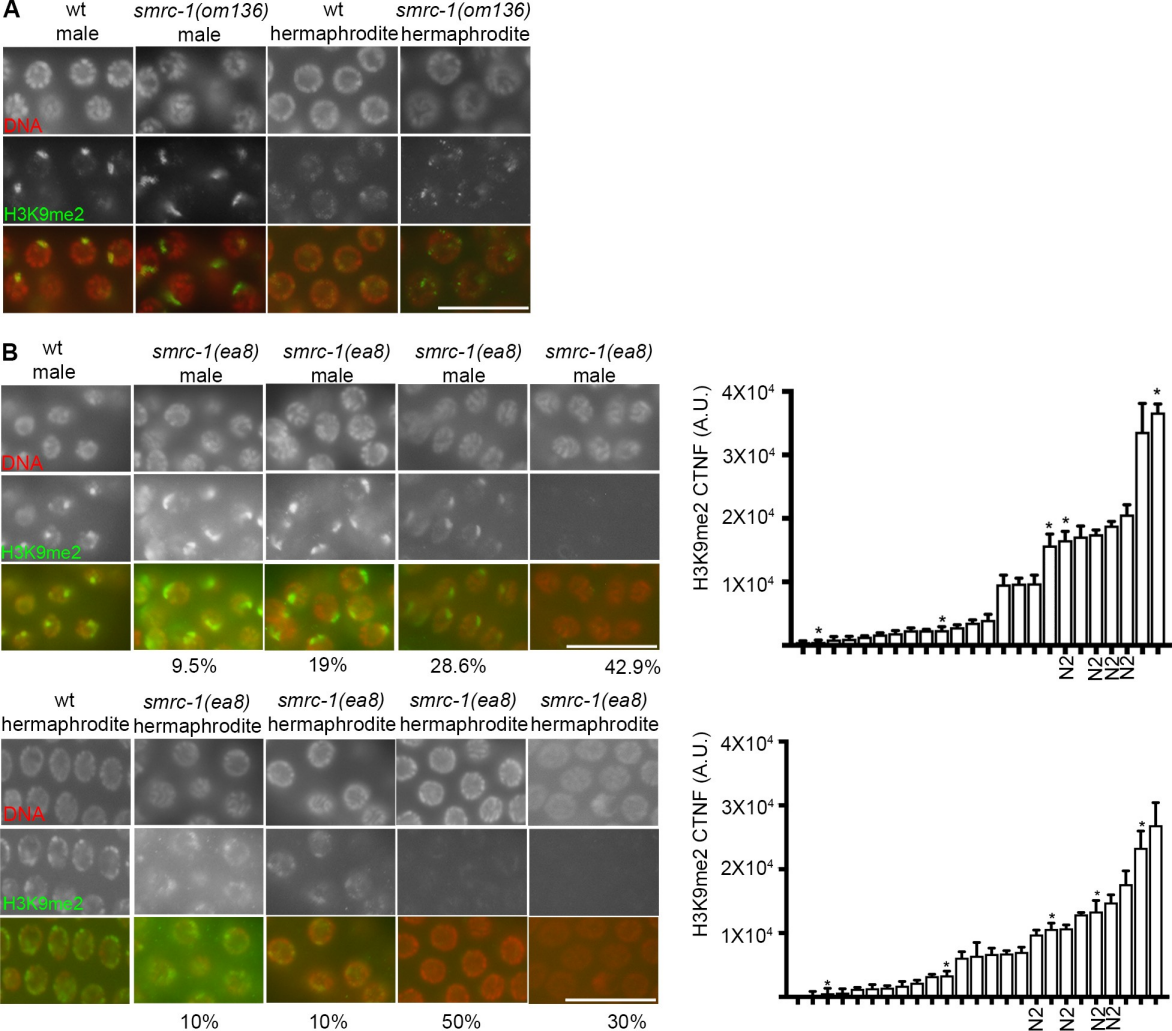
H3K9me2 labeling is reduced in serially passaged *smrc-1* mutants. (A) H3K9me2 distribution as detected by immunolabeling in wildtype and *smrc-1(om136)* F2 worms raised at 25°C. Gonads were dissected at 18 hr post-L4 stage and labeled with anti-H3K9me2 antibody and counterstained with DAPI to visualize DNA. Panel show sets of pachytene nuclei. (B) H3K9me2 distribution as detected in wildtype and *smrc-1(ea8)* animals passaged for 30 generations at 25°C. Gonads were dissected at 18 hr post-L4 stage and labeled with anti-H3K9me2 antibody and counterstained with DAPI to visualize DNA. Each panel shows a set of pachytene nuclei from four different individuals with different staining intensities and/or patterns. Histograms indicate the corrected total nuclear fluorescence (CTNF, see Materials and methods) for a set of nuclei within randomly selected wildtype and *smrc-1* F30 germ lines; asterisks indicate the germ lines included in the images. Number of gonads assayed: 4 wt and 21 *smrc-1* males; 4 wt and 20 *smrc-1* hermaphrodites. 6 germline nuclei assayed/gonad.

We asked whether SMRC-1 promotes germline H3K9me2 by immunolabeling *smrc-1* mutants passaged for either two or 30 generations at 25°C. We evaluated *smrc-1(om136)* XX hermaphrodite and XO male germlines in the M-Z- F2 generation and in five *smrc-1(ea8)* lines in the F30 generation. In M-Z- F2 animals, the H3K9me2 labeling pattern appeared comparable to wild type in both *smrc-1* XO and XX germ cells (Fig 9A). Among F30 germ lines, the average H3K9me2 signal was weaker than wildtype in a majority of gonads evaluated (Fig 9B). In a small subset, H3K9me2 signal was comparable to or greater than controls (Fig 9B). H3K9me2 signal was similar among nuclei within individual germ lines, suggesting a systemic change in H3K9me2 regulation throughout the tissue.

### Loss of *met-2* function enhances *smrc-1* sterility

To investigate the genetic relationship between *smrc-1* and *met-2*, we generated a *smrc-1 met-2* double knockout strain (S1A Fig) and assayed the *smrc-1 met-2* phenotype in parallel with *met-2* and *smrc-1* single mutants (Tables 1 and 2). At 25°C, *met-2* and *smrc-1* homozygotes remained fertile for numerous generations. In contrast, *smrc-1 met-2* double mutant fertility dropped to near zero by the F2 generation. The clutch size of *smrc-1 met-2* M+Z- F1 double mutants was similar to *smrc-1* M+Z- F1 single mutants, but a lower proportion of offspring were viable (Table 1). Only 8% of the viable *smrc-1 met-2* M-Z- offspring were fertile (Table 2), and they produced very few embryos, only ~6% of which were viable (Table 1). We also observed a protruding vulva (Pvl) phenotype in ~43% of *smrc-1 met-2* M-Z- animals (Table 2) that may reflect DNA damage in the vulval precursor cells [77]. Overall, the *smrc-1 met-2* phenotype is consistent with SMRC-1 and MET-2 acting redundantly to promote one or more essential germline process(es).

#### Gonad development

To investigate germline defects underlying *smrc-1 met-2* sterility, we DAPI-stained F2 M-Z- hermaphrodites and evaluated CED-1::GFP expression. Germ cells were present in 100% of gonad arms examined (S2 Fig). ~76% of gonad arms had normal U-shaped morphology; the germ cell populations in most of these arms produced sperm, and many also produced oocytes. However, somatic gonad development was defective in ~24% of gonad arms; in these cases, germ cells were clustered in the vicinity of the vulva, and no gametes were present. In analyzing CED-1::GFP, we looked at *met-2* single mutants as well as *smrc-1 met-2*. Interestingly, *met-2* single mutants had significantly more apoptotic germ cells than wildtype (Fig 3A). Among *smrc-1 met-2* F2 M-Z- germ lines, we observed three patterns of CED-1::GFP expression. When the somatic gonad had developed normally, we observed apoptosis throughout the germ line (Fig 3A). This phenotype resembles sterile *smrc-1* mutants and is more severe than fertile *smrc-1* mutants (Fig 3A). When the gonad arm had not extended and germ cells were clustered in the proximal gonad, CED-1::GFP-positive cells were present in that proximal region in some cases (S2C Fig). A third pattern was absence of CED-1::GFP expression, perhaps reflecting the inability to activate the DNA damage response or a sheath cell defect (S2C Fig). We conclude the *smrc-1 met-2* sterility results from (i) somatic gonad defects, (ii) failure to produce oocytes, or (iii) failure to produce fertilization-competent gametes, depending on the individual. In most cases, extensive germline apoptosis occurred.

#### DNA replication stress

We wanted to determine if MET-2 and SMRC-1 function in a common mechanism to limit DNA replication stress. If MET-2 and SMRC-1 act in parallel to limit replication stress, then we expect there to be additive or synergistic effect of HU treatment. This effect might contribute to the *smrc-1 met-2* sterility. In contrast, if they work in a common mechanism, then we expect little or no change in HU sensitivity in the double mutant compared to single mutants. We assayed HU sensitivity of *smrc-1 met-2* M+Z- double mutants using the L1 treatment regimen described above (Fig 1B) and compared it to *smrc-1* M+Z- and *smrc-1* M-Z- as well as *met-2* and wild-type sensitivities. Loss of *met-2* alone increased sensitivity to HU across all doses (Fig 1A). The impact of HU treatment on *smrc-1 met-2* viability was more complex. Viability of *smrc-1 met-2* was not significantly different from *smrc-1* mutants at 2.5 and 5 mM HU, but was significantly worse at 10 and 25 mM HU (Fig 1B). In contrast, sterility was not significantly higher in the *smrc-1 met-2* M+Z- double mutants compared with *smrc-1* or *met-2* controls at any dose except 10 mM (Fig 1B). Overall, these results are consistent with SMRC-1 and MET-2 acting in a common pathway to protect against replication stress in the germ line. Overall, MET-2 appears to provide protection from replication stress in the soma, and this protection may involve MET-2 acting both in concert with and independent of SMRC-1.

#### Poly G/C tract integrity

To investigate if a higher mutation rate contributes to the *smrc-1 met-2* phenotype, we screened for enhancement of the *dog-1* phenotype in *met-2* single and *smrc-1 met-2* double mutant backgrounds using the *vab-1* exon 5 deletion assay described above. The deletion frequency at 25°C was four-fold higher in *dog-1;met-2* double mutants compared to *dog-1* single mutants assayed in parallel (F3 generation, p<0.0001) and nearly three-fold higher in *dog-1;smrc-1 met-2* M+Z- triple mutants compared to *dog-1* single mutants (Fig 3C, p<0.01). The frequency of mutations at the *vab-1* locus was not statistically different between *dog-1;smrc-1 met-2* and either *dog-1;met-2* or *dog-1;smrc-1*. Together, these results suggest that MET-2 limits accumulation of deletions in poly G/C regions in the absence of DOG-1 activity, and that SMRC-1 and MET-2 act together for this function.

### The relationship between SMRC-1 and SET-25 activity

Given that MET-2 and SET-25 modify some common regions of the genome, we considered that SET-25 activity might contribute to SMRC-1-related processes. To address this question, we first investigated the impact of SET-25 loss on the *smrc-1* developmental phenotype at 25°C. 100% of *set-25* single mutants were fertile. ~8% of *smrc-1 set-25* double mutants were sterile, similar to *smrc-1* single mutants, and the two genotypes had similar developmental defects (Table 2, S2B Fig). We next investigated the impact of SET-25 loss on HU sensitivity. The response *set-25* single mutants to HU resembled wild type and response of *smrc-1 set-25* double mutants resembled *smrc-1* single mutants (Fig 1). We conclude that SET-25 activity is not essential for protection from DNA replication stress, and loss of SET-25 activity does not impact the *smrc-1* developmental phenotype.

The *met-2(n4256) set-25(tm5021)* double mutant was previously described as slow growing with substantial embryonic lethality, elevated HU sensitivity, and increased CEP-1-dependent germline apoptosis at 25°C [17]. *met-2 set-25* double mutants also produce some abnormal oocytes and have elevated apoptosis [18]. We regenerated the *met-2(n4256) set-25(tm5021)* double mutant and grew it in parallel with *smrc-1 met-2* and *smrc-1 set-25* to compare germline development of animals grown together under the same conditions. At 25°C, embryonic lethality was very high in *met-2 set-25* double mutants and all adult escapers were fertile, as reported (Table 2).

We also evaluated HU sensitivity in the *met-2 smrc-1 set-25* triple mutant. Since sterility was high in the *met-2 smrc-1 set-25* M-Z- F2 individuals, we analyzed the responsiveness of M+Z- F1 individuals to HU exposure (Fig 1B). At the highest doses of HU, *met-2 smrc-1 set-25* M+Z- sensitivity was significantly elevated compared to either *smrc-1 met-2* or *smrc-1 set-25* M+Z- double mutants and the effect on fertility (i.e., the effect in the germ line) was particularly striking (Fig 1B). Hence, H3K9 methylation *per se* combined with SMRC-1 together provide substantial protection from replication stress.

## Discussion

Chromatin structure is carefully modulated to limit DNA damage and maintain genome integrity. Our analysis identifies a link between conserved components of the DNA repair and chromatin regulatory machineries in *C*. *elegans*: SMRC-1, a member of the SMARCAL1 annealing helicase family known to promote DNA repair during replication; and MET-2, a member of the SETDB1 histone methyltransferase family responsible for H3K9 methylation. By a variety of measures, we show that SMRC-1 promotes germline viability and limits DNA damage. Nuclear SMRC-1 abundance increases in the proliferative germ line under conditions of replication stress, and SMRC-1 promotes a concomitant increase in nuclear MET-2 accumulation. There is a small, but statistically significant increase in detectable H3K9me2 signal in these nuclei. When SMRC-1-deficient mutants are maintained long-term at elevated culture temperatures, H3K9me2 deposition becomes unregulated, and in most cases reduced. Fertility defects arise in these serially passaged *smrc-1* mutants, which we hypothesize result from accumulated DNA damage over multiple generations due to chronic replication stress and reduced H3K9me2 deposition.

Two observations suggest that H3K9me2 limits the negative consequences of replication stress in the germ line: HU treatment causes an increase in nuclear MET-2 accumulation; and *met-2* mutants are hypersensitive to HU with respect to fertility. In our assays, MET-2 limits replication stress independently of SET-25, suggesting H3K9 mono- and di-methyl marks are more important than trimethyl marks in this context. We also demonstrate a previously unappreciated role for MET-2 in stabilizing poly G/C tracts.

### SMRC-1 limits DNA damage

SMRC-1 promotes fertility, likely as a consequence of its roles in DNA repair and limiting DNA damage. We hypothesize that the increased sensitivity to replication stress and reduced ability to repair DNA lesions contribute to the *smrc-1* developmental phenotypes. SMRC-1 is particularly important at elevated culture temperatures, and we note that the loss-of-function phenotypes of *Drosophila* Marcal1 and mouse SMARCAL are also more severe at elevated culture temperature [78]. SMRC-1 buffers against replication stress, limits R-loop accumulation, limits SPO-11-independent DSBs, promotes MET-2 accumulation in the nucleus under conditions of replication stress, and affects the distribution of meiotic crossovers. At stressful temperatures, SMRC-1 activity affects H3K9me2 accumulation throughout the germ line. The association with SMRC-1 may recruit MET-2 to the nucleus where they may function at the replication fork. SMARCAL1 family proteins are hypothesized to function outside of S phase to promote DSB repair [79, 80]. and SMRC-1 may recruit MET-2 to help stabilize chromatin for repair in this context.

### Alternative models for cooperation between MET-2 –SMRC-1

Based on genetic data, SMRC-1 and MET-2 appear to both shared and distinct functions in the germ line. We hypothesize that SMRC-1 and MET-2 act together to limit germline sensitivity to replication stress. In contrast, the *met-2 smrc-1* synthetic sterility may be the cumulative effect of severely reduced H3K9 methylation in combination with DNA damage beyond that at replication forks.

**Fig 10:**
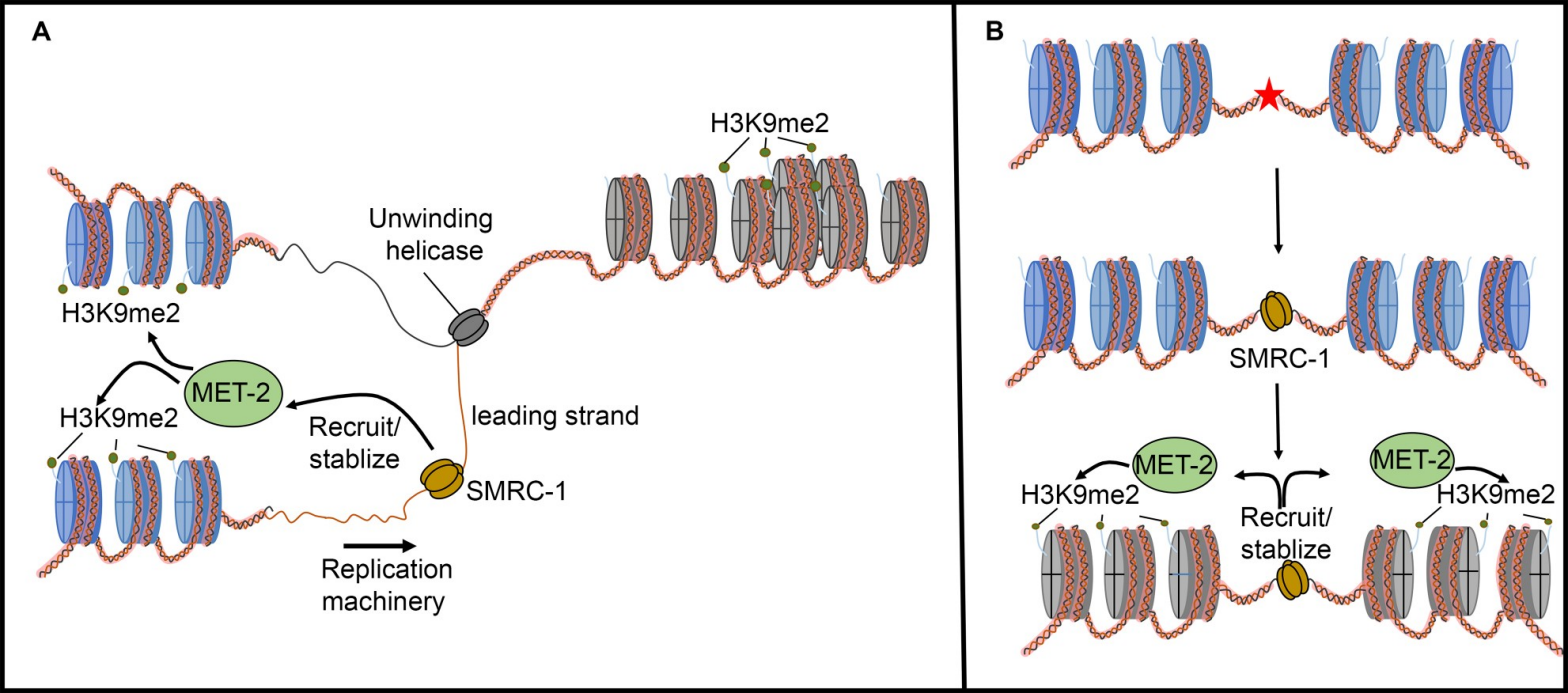
Alternative models for the relationship between MET-2 and SMRC-1 activity. Association of MET-2 with SMRC-1 may promote deposition of H3K9 methylation (A) on nascent chromatin following DNA replication and/or (B) at sites of DNA damage (star). This second alternative may occur during DNA replication and/or at other times in the cell cycle (as depicted). In this scenario, SMRC-1 is recruited to the damaged site to facilitate the repair of a DNA lesion. MET-2 associates with SMRC-1 and methylates H3K9. Deposition of H3K9me2 and establishment of heterochromatin near a DNA break site stabilizes the exposed break until repair occurs.

We consider two non-mutually exclusive models for how the MET-2 –SMRC-1 association may promote genome integrity. First, SMARCAL1 family proteins associate with ssDNA at the replication fork, hence SMRC-1 may be well-positioned to recruit MET-2 for re-establishment of H3K9me2 marks on nascent chromatin (Fig 10A). Reestablishing heterochromatin at repetitive sequences after DNA replication is important for maintaining genome stability [81–83]. Second, SMRC-1 may recruit MET-2 to DNA breaks, thereby stabilizing DNA and facilitating repair. The interaction could be important when breaks arise during replication and/or at another point in the cell cycle (Fig 10B). SETDB1 is recruited to DNA damage sites directly and specifically in mammalian systems and SETDB1 enrichment is essential for proper repair of the DNA lesions [84]. Consistent with this finding, Checchi et al. (2011) observed elevated germline apoptosis and sensitivity to *cep-1* loss in animals treated with *met-2* RNAi, which may indicate a role for MET-2 in mediating the DNA damage response [85]. At repetitive regions, MET-2-mediated H3K9me2 deposition may have an additional beneficial effect of reducing the DNA replication rate to assure the complete and accurate replication of error-prone repetitive sequences. In this scenario, MET-2 may function in a positive feed-back loop to attract more SMRC-1, thus reinforcing replication fidelity. It has been proposed that MET-2-mediated H3K9me2 deposition limits transcription of repetitive regions and thereby limits RNA:DNA hybrid formation at those sites [17]. Perhaps one way in which MET-2 limits RNA:DNA hybrid stability is by recruiting SMRC-1, which may have a role in resolving the hybrids.

### SMRC-1 and meiosis

Little is known about possible meiotic functions for SMARCAL1 family proteins as functional studies have only been performed in mitotic cells. Mammalian SMARCAL1 associates with the ssDNA binding protein, RPA, during DNA replication and catalyzes replication fork regression, ultimately promoting branch migration [38, 43, 86, 87]. Holliday junctions, which resemble replication forks, are present during meiotic recombination, and *C*. *elegans* RPA (RPA-1) is present in pachytene nuclei and promotes meiotic DSB repair [88, 89]. The meiotic recombination pattern observed in *smrc-1* mutants may therefore have multiple underlying causes.

*C*. *elegans* meiotic DSBs are enriched on chromosomal arms where they inversely correlate with repetitive sequences and H3K9me2 enrichment [71, 90]. These data fit with observations from a number of species that DSBs–and therefore COs–tend not to occur at repetitive sequences, perhaps in part due to H3K9me2 [2]. Our RAD-51 labeling data and diakinesis chromosome analyses indicate that SMRC-1 protects the genome from aberrant DSBs and inaccurate repair. In *smrc-1* mutants, RAD-51 foci persisted late into pachytene, consistent with delayed DSB repair in the absence of SMRC-1. In *C*. *elegans*, the process of CO homeostasis ensures that most DSBs are repaired via a non-crossover (NCO) mechanism and only one DSB per chromosome is resolved via CO [67]. Our mapping data indicate that SMRC-1 activity promotes CO homeostasis. One explanation for the loss of CO homeostasis in *smrc-1* mutants may be that aberrant DSBs in the *smrc-1* proliferative germ line are not subject to the same strict regulatory controls as SPO-11-induced breaks. An alternative hypothesis is that SMRC-1 activity limits CO frequency.

Human SMARCAL1 promotes DSB repair via non-homologous end joining (NHEJ) in cultured cells [80] and *Drosophila* Marcal1 mediates the synthesis-dependent strand annealing (SDSA) step in DSB DNA repair [79]. SMRC-1 activity may limit meiotic recombination by promoting NCO repair, perhaps by recruiting/stabilizing MET-2 at repetitive regions. The association between MET-2 and SMRC-1 could serve as a surveillance system to prevent DSB formation at repetitive regions, thus limiting the occurrence of CO at these sequences.

## Materials and methods

### Ethics statement

Syracuse University issued an IACUC number to E.M.M. for the custom anti-MET-2 antibody generation, which was performed by Yenzym Antibodies LLC. The Syracuse University IACUC number is #09 = 021.

### Nematode strains and maintenance

*C*. *elegans* were maintained according to standard methods [91]. Details of nematode strains, mutant construction by CRISPR, and epitope tagging can be found in S1 Text.

### Protein blots and immunolabeling

Protein blots and immunohistochemistry were performed using standard methods. Detailed procedures, including antibodies used and quantification methods, can be found in S1 Text.

### Immunoprecipitation

MET-2 IP was performed with nuclear extract prepared from *him-8(e1489)* adults. 3xFLAG::SMRC-1 IP was performed with whole extract from endogenously-tagged *3xflag*::*smrc-1* adults. Detailed procedures can be found in S1 Text.

### HU assay

Assays were carried put as previously described [92]. L1 larvae of different genotypes were treated with HU for a pulse of 16 hr at 25°C and then cultured using standard conditions. L4 larvae were treated with HU for 16 hr at room temperature (~22°C) until adulthood, and then immunolabeled. Detailed HU treatment protocols can be found in S1 Text.

### *unc-58* suppression and *dog-1* enhancement

We assayed suppression/reversion of the *unc-58(e665)* phenotype as described [59] in *unc-58* control and *smrc-1(ea8);unc-58* mutants raised at 20°C. To detect *dog-1* enhancement, we assayed for deletions in *vab-3* exon 5 as described [62]. Details are included in S1 Text.

### Transgenerational broods and sterility

Six lines of balanced *smrc-1(ea8)/qC1* were maintained at 25°C for three generations and then expanded to 16 unbalanced founders. Strains were maintained by serial passaging as described in the S1 Text.

## Supporting information

S1 Fig Antiserum and transgenes generated for analysis of MET-2 and SMRC-1. (A) Locations of epitope tags and mutant lesions generated via CRISPR. Boxes and lines represent exons and introns, respectively. Black arrows indicate predicted Cas9 cutting site for each injected sgRNA. *smrc-1(om136)* contains a stop codon inserted in-frame at the 5^th^ codon in exon 1. *smrc-1(om138)* is a frameshift allele generated by inserting two nucleotides at codon 7 of exon 1. *smrc-1(ea8)* and *smrc-1(ea46)* are deletions, as indicated. *smrc-1(ea173)* was generated in the polymorphic CB4856 background; contains two nucleotide substitutions and a deletion, as indicated. *3xflag*::*smrc-1* and *3xflag*::*met-2* were generated by in-frame insertion of *3xflag* coding sequences immediately after the start codon. See Experimental Procedures.
(B) Wildtype (strain N2) tissue serves as a negative control for anti-FLAG immunolabeling. (C) Immunolabeling of H3K9me2 in N2 wildtype and CRISPR-tagged *3xflag*::*met-2* (strain EL634) germlines. Dissected gonads are oriented with distal end to the left. DNA was visualized with DAPI. (D) Dissected *met-2(+);him-8(e1489)* and *met-2(n4256)* adult male gonads were immunolabeled with anti-MET-2 antibody and counterstained with DAPI to visualize DNA. Pachytene nuclei are shown. Nuclear signal is not detected in *met-2(n4256)* tissue. Scale bar: 16 μm. (E) Broods of wildtype, *met-2(n4256)*, and *omIs1[met-2*::*gfp]* lines at 20°C. *omIs1* rescues the *met-2(n4256)* brood size.
(TIF)

S2 Fig Germline defects observed in *smrc-1* and *met-2 smrc-1* M-Z- mutants at 25°C. (A) Distribution of germline defects in F2 M-Z- mutants. N, number of sterile gonad arms evaluated. Note that sterile hermaphrodites represent only ~8% of the total *smrc-1* M-Z- population and a much larger 92% of the *met-2 smrc-1* M-Z- population. *, Includes all individuals with somatic gonad defects. (B) Examples of adult mutant hermaphrodites labeled with the DNA dye, DAPI, to visualize germ cell morphology. Relevant germline features are labeled. *, distal end of gonad arm. (C) CED-1::GFP expression in adult *smrc-1 met-2* M-Z- hermaphrodite germ lines. Images show representative examples of the three different CED-1::GFP expression patterns in *smrc-1 met-2* M+Z- individuals raised at 25°C. Upper panels, differential contrast interference (DIC) images. Lower panels, GFP expression. Left, the gonad arm failed to extend, and a small cluster of germ cells is present adjacent to the vulva. Arrow, proximal germ cells undergoing engulfment. Middle, CED-1::GFP is present throughout the gonad arm indicating extensive apoptosis. Right, CED-1::GFP is not visible. N = 54.
(TIF)

S3 Fig SMRC-1 impacts the distribution of RAD-51 foci and crossover events. (A) SMRC-1 activity impacts the distribution of RAD-51 foci during meiotic prophase. Data are summarized for wildtype and *smrc-1*, *spo-11* and *smrc-1;spo-11* mutants. Diagram represents a hermaphrodite germline where the nuclei in leptotene–pachytene have been evenly divided into six zones based on cell row counts. The key indicates the percentage of total nuclei containing the indicated number of RAD-51 foci. (B) Recombination frequency was mapped in two genetic intervals in the chromosome I gene cluster defined by *unc-11 dpy-5* (genetic map position -2.51 to 0.00) and *dpy-5 unc-13* (genetic map position 0.00 to +2.07). Wildtype and *smrc-1(om136)* animals were assayed in parallel. Recombination frequency was calculated according to Brenner [91]. (C) Whole chromosome I mapping detected an ~7.4-fold increase in double recombination events in *smrc-1(-)* relative to wildtype. (D) Overall crossover distribution in *smrc-1* mutants resembles wildtype except in interval 4. * P<0.03. Data are presented as % (number of events).
(TIF)

S4 Fig SMRC-1 and MET-2 localize to mitotic and pachytene germline nuclei. Germline tissue co-labeled with anti-MYC and anti-FLAG, counterstained with DAPI, and visualized with confocal microscopy. Pairwise combinations of DNA, MET-2, and SMRC-1 labeling are shown for (A) pachytene and (B) proliferative germ cells. Note that (A) includes the same tissue shown without DNA labeling in Fig 6C. Single-label images are shown in grey scale. Merged images: 3xFLAG::MET-2 (red), 3xMYC::SMRC-1 (green), DNA (blue). Scale bar: 5 μm. Arrows indicate example of regions with co-labeling.
(TIF)

S5 Fig SMRC-1 signal in distal germline nuclei normalized to histone H3. SMRC-1 abundance in distal germ cell nuclei increases upon exposure to hydroxyurea. Box-and-whisker plots represent the mean anti-FLAG immunolabeling intensity as normalized to (left) the mean DAPI fluorescence intensity and (right) the mean anti-H3 fluorescence intensity. These data complement and are consistent with normalization data presented in Fig 7A. For each mitotic zone, 5–7 nuclei in a similar state of chromatin condensation and a single focal plane were measured; 6–8 germlines were measured per biological replicate per genotype. Scale bar, 16 μm.
(TIF)

S1 Table *smrc-1* M+Z- are viable at 25°C. (DOCX)

S2 Table Acridine orange quantification of germline apoptotic bodies at 25°C. (DOCX)

S1 Text Supplemental Materials and methods and References. (DOCX)

S1 Data Numeric data for figures. Spreadsheet contains the numeric data for graphs and statistics contained in Figs 1, 2, 3, 7, 8, 9, S1, S3, and S5. Data for each figure are included on a separate page of the spreadsheet.
(XLSX)
