# Supplementary figures and images for: A DNA repair protein and histone methyltransferase interact to promote genome stability in the Caenorhabditis elegans germ line
Source: PLoS Genet. 2019 Feb 22;15(2):e1007992. doi: 10.1371/journal.pgen.1007992 (PMC6402707; doi:10.1371/journal.pgen.1007992)

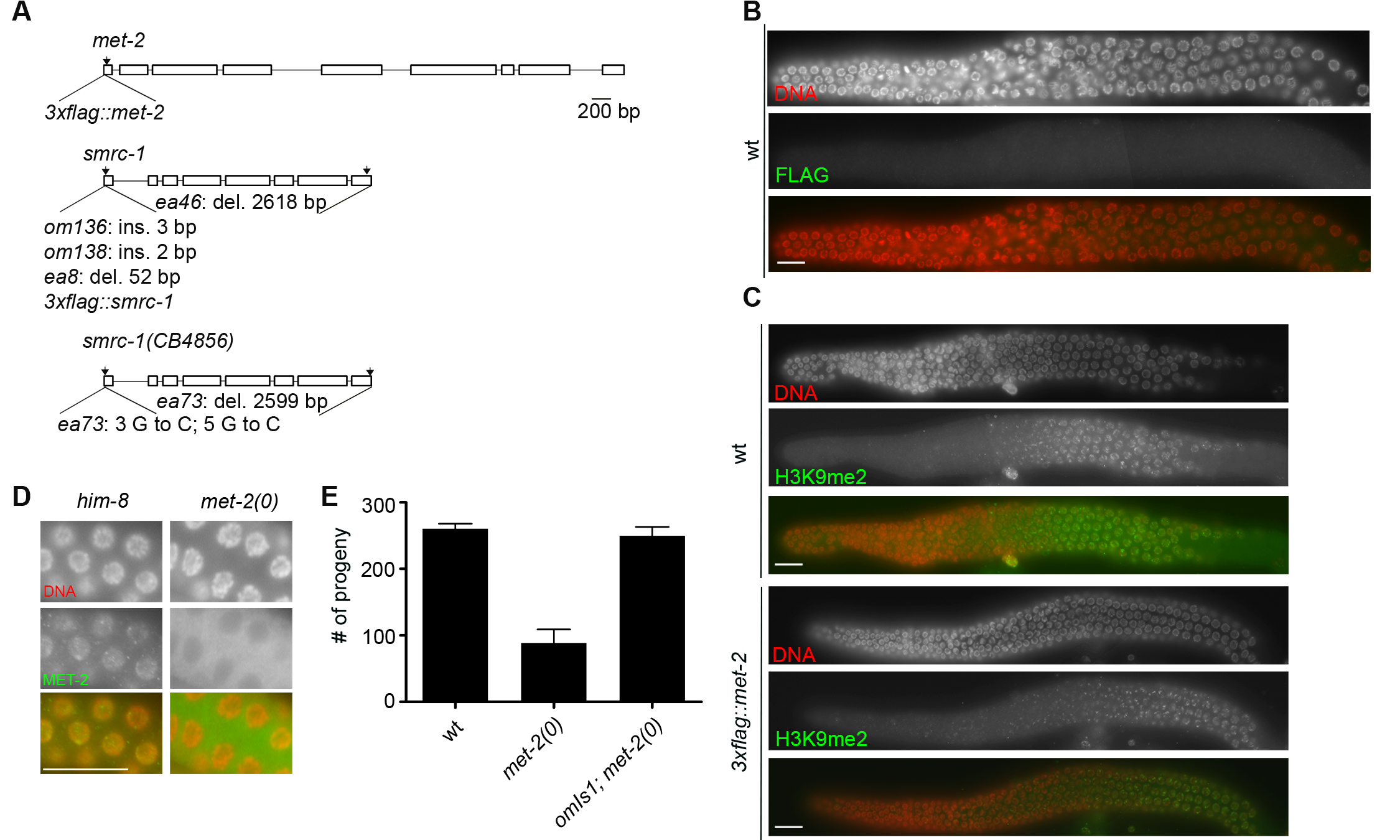

Supplement: S1 Fig — (A) Locations of epitope tags and mutant lesions generated via CRISPR. Boxes and lines represent exons and introns, respectively. Black arrows indicate predicted Cas9 cutting site for each injected sgRNA. smrc-1(om136) contains a stop codon inserted in-frame at the 5th codon in exon 1. smrc-1(om138) is a frameshift allele generated by inserting two nucleotides at codon 7 of exon 1. smrc-1(ea8) and smrc-1(ea46) are deletions, as indicated. smrc-1(ea173) was generated in the polymorphic CB4856 background; contains two nucleotide substitutions and a deletion, as indicated. 3xflag::smrc-1 and 3xflag::met-2 were generated by in-frame insertion of 3xflag coding sequences immediately after the start codon. See Experimental Procedures. (B) Wildtype (strain N2) tissue serves as a negative control for anti-FLAG immunolabeling. (C) Immunolabeling of H3K9me2 in N2 wildtype and CRISPR-tagged 3xflag::met-2 (strain EL634) germlines. Dissected gonads are oriented with distal end to the left. DNA was visualized with DAPI. (D) Dissected met-2(+);him-8(e1489) and met-2(n4256) adult male gonads were immunolabeled with anti-MET-2 antibody and counterstained with DAPI to visualize DNA. Pachytene nuclei are shown. Nuclear signal is not detected in met-2(n4256) tissue. Scale bar: 16 μm. (E) Broods of wildtype, met-2(n4256), and omIs1[met-2::gfp] lines at 20°C. omIs1 rescues the met-2(n4256) brood size. (TIF) [file pgen.1007992.s001.tif]

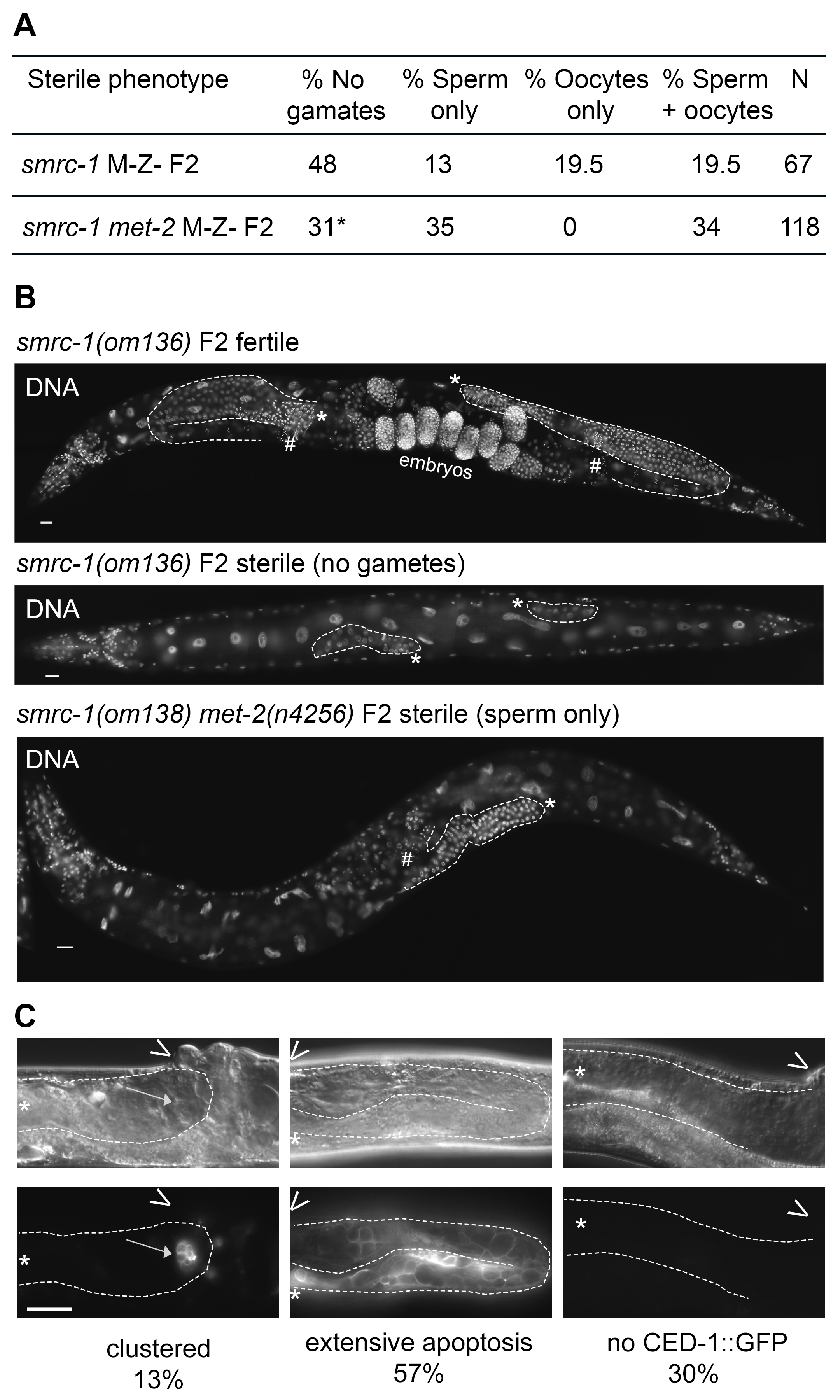

Supplement: S2 Fig — (A) Distribution of germline defects in F2 M-Z- mutants. N, number of sterile gonad arms evaluated. Note that sterile hermaphrodites represent only ~8% of the total smrc-1 M-Z- population and a much larger 92% of the met-2 smrc-1 M-Z- population. *, Includes all individuals with somatic gonad defects. (B) Examples of adult mutant hermaphrodites labeled with the DNA dye, DAPI, to visualize germ cell morphology. Relevant germline features are labeled. *, distal end of gonad arm. (C) CED-1::GFP expression in adult smrc-1 met-2 M-Z- hermaphrodite germ lines. Images show representative examples of the three different CED-1::GFP expression patterns in smrc-1 met-2 M+Z- individuals raised at 25°C. Upper panels, differential contrast interference (DIC) images. Lower panels, GFP expression. Left, the gonad arm failed to extend, and a small cluster of germ cells is present adjacent to the vulva. Arrow, proximal germ cells undergoing engulfment. Middle, CED-1::GFP is present throughout the gonad arm indicating extensive apoptosis. Right, CED-1::GFP is not visible. N = 54. (TIF) [file pgen.1007992.s002.tif]

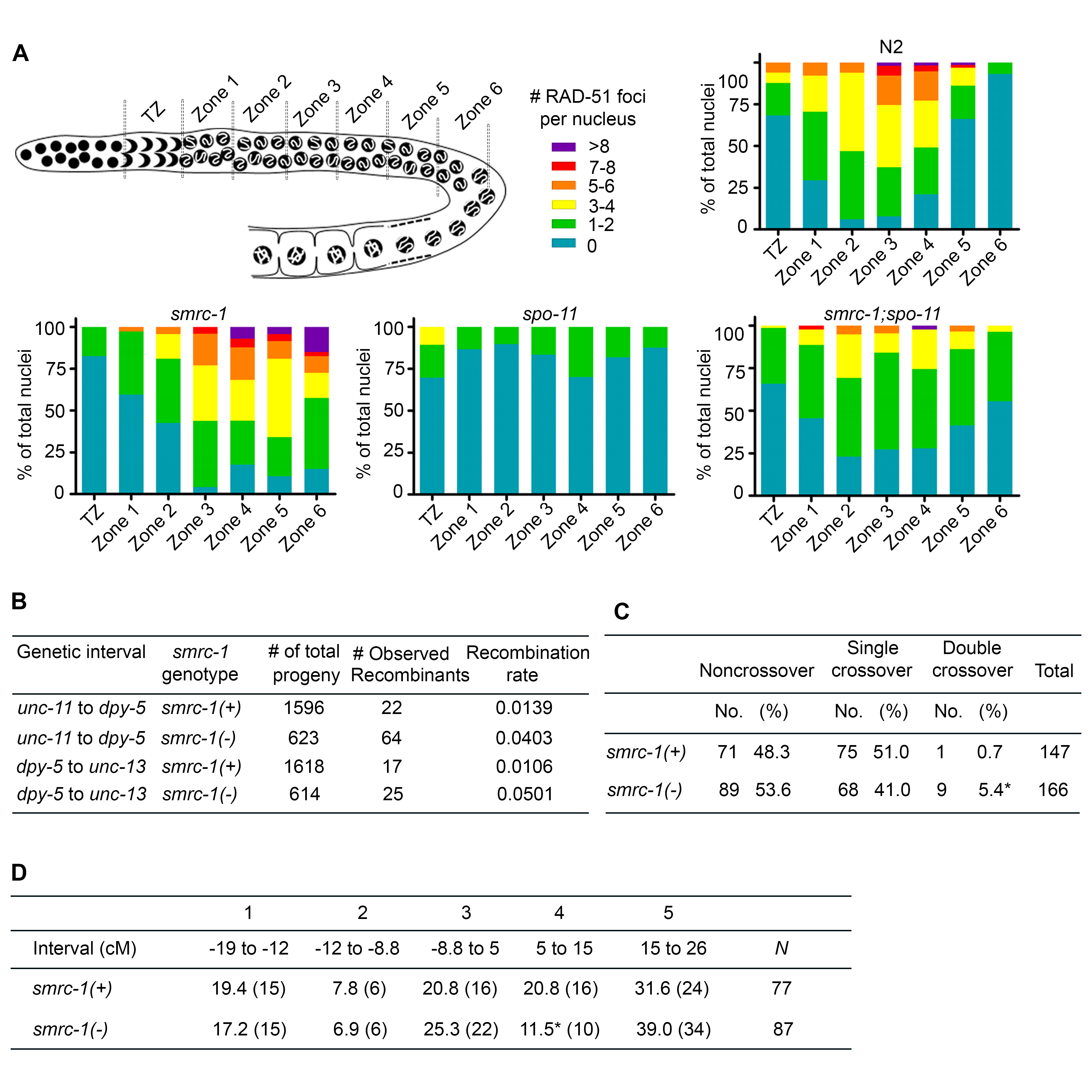

Supplement: S3 Fig — (A) SMRC-1 activity impacts the distribution of RAD-51 foci during meiotic prophase. Data are summarized for wildtype and smrc-1, spo-11 and smrc-1;spo-11 mutants. Diagram represents a hermaphrodite germline where the nuclei in leptotene–pachytene have been evenly divided into six zones based on cell row counts. The key indicates the percentage of total nuclei containing the indicated number of RAD-51 foci. (B) Recombination frequency was mapped in two genetic intervals in the chromosome I gene cluster defined by unc-11 dpy-5 (genetic map position -2.51 to 0.00) and dpy-5 unc-13 (genetic map position 0.00 to +2.07). Wildtype and smrc-1(om136) animals were assayed in parallel. Recombination frequency was calculated according to Brenner [91]. (C) Whole chromosome I mapping detected an ~7.4-fold increase in double recombination events in smrc-1(-) relative to wildtype. (D) Overall crossover distribution in smrc-1 mutants resembles wildtype except in interval 4. * P<0.03. Data are presented as % (number of events). (TIF) [file pgen.1007992.s003.tif]

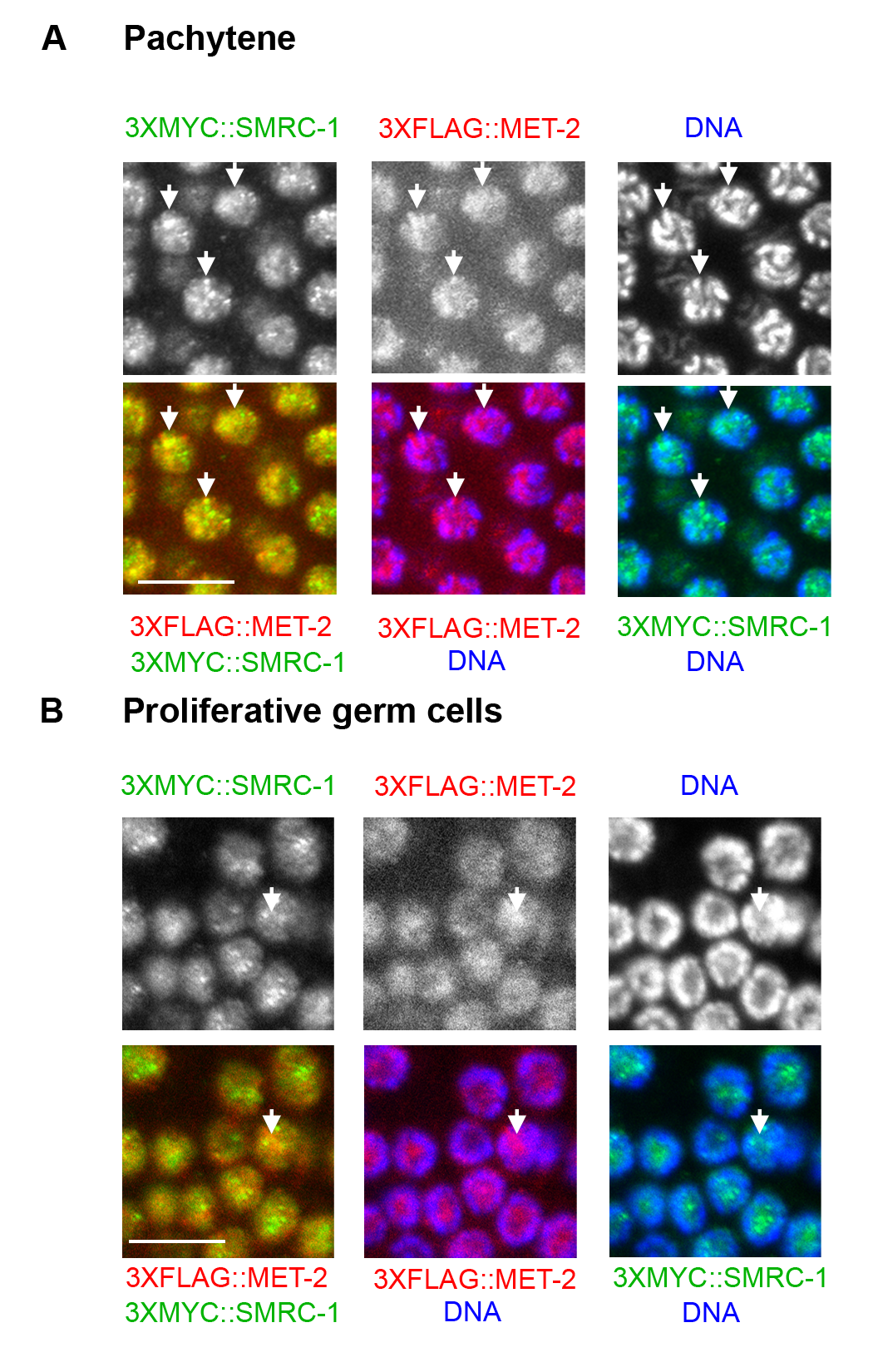

Supplement: S4 Fig — Germline tissue co-labeled with anti-MYC and anti-FLAG, counterstained with DAPI, and visualized with confocal microscopy. Pairwise combinations of DNA, MET-2, and SMRC-1 labeling are shown for (A) pachytene and (B) proliferative germ cells. Note that (A) includes the same tissue shown without DNA labeling in Fig 6C. Single-label images are shown in grey scale. Merged images: 3xFLAG::MET-2 (red), 3xMYC::SMRC-1 (green), DNA (blue). Scale bar: 5 μm. Arrows indicate example of regions with co-labeling. (TIF) [file pgen.1007992.s004.tif]

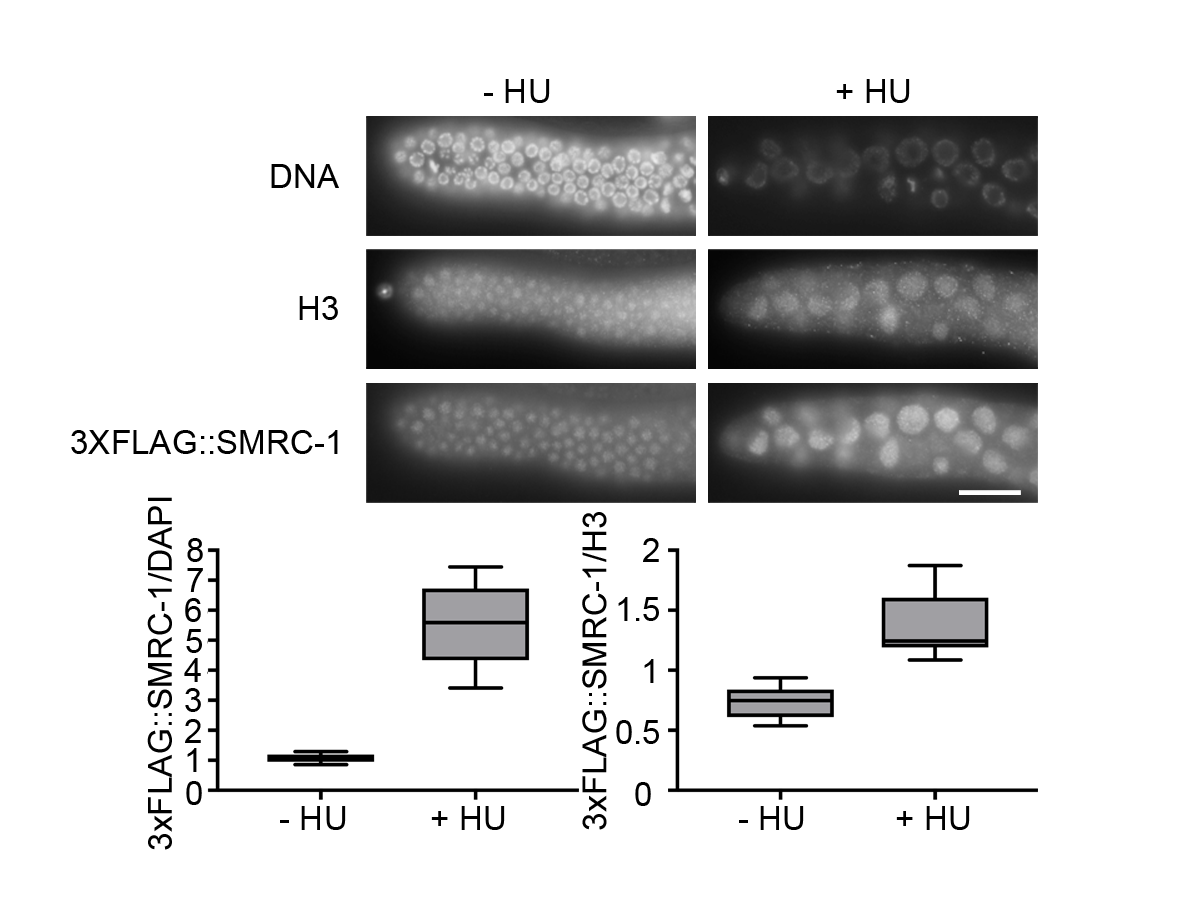

Supplement: S5 Fig — SMRC-1 abundance in distal germ cell nuclei increases upon exposure to hydroxyurea. Box-and-whisker plots represent the mean anti-FLAG immunolabeling intensity as normalized to (left) the mean DAPI fluorescence intensity and (right) the mean anti-H3 fluorescence intensity. These data complement and are consistent with normalization data presented in Fig 7A. For each mitotic zone, 5–7 nuclei in a similar state of chromatin condensation and a single focal plane were measured; 6–8 germlines were measured per biological replicate per genotype. Scale bar, 16 μm. (TIF) [file pgen.1007992.s005.tif]
