# Supplementary material for: A DNA repair protein and histone methyltransferase interact to promote genome stability in the Caenorhabditis elegans germ line
Source: PLoS Genet. 2019 Feb 22;15(2):e1007992. doi: 10.1371/journal.pgen.1007992 (PMC6402707; doi:10.1371/journal.pgen.1007992)
Supplement: S1 Table — (DOCX) [file pgen.1007992.s006.docx]

**S1 Table** *smrc-1* M+Z- are viable at 25°C

| Genotype | N | Avg clutch + SEM | % Viable progeny | # Fertile *smrc-1* M+Z- | # Sterile *smrc-1* M+Z- |
| --- | --- | --- | --- | --- | --- |
| *smrc-1(om136)/qC1[gfp]* | 5 | 148 + 7 | 96 | 34 + 2 | 0 |
| *smrc-1(om138)/qC1[gfp]* | 4 | 136 + 1 | 96 | 32 + 0.3 | 0 |

N, number of clutches quantified. *smrc-1* M+Z- individuals are identified by the absence of GFP expression. *qC1[gfp]* heterozygotes produce ~95% viable progeny [1]. N, number of clutches quantified.
