# Supplementary material for: A DNA repair protein and histone methyltransferase interact to promote genome stability in the Caenorhabditis elegans germ line
Source: PLoS Genet. 2019 Feb 22;15(2):e1007992. doi: 10.1371/journal.pgen.1007992 (PMC6402707; doi:10.1371/journal.pgen.1007992)
Supplement: S2 Table — (DOCX) [file pgen.1007992.s007.docx]

**S2 Table** Acridine orange quantification of germline apoptotic bodies at 25°C

| Genotype | Avg # AO-stained bodies | SEM | N |
| --- | --- | --- | --- |
| N2 wt | 5.0 | 0.50 | 20 |
| *smrc-1(ea8)* | 7.87^***^ | 0.47 | 38 |
| *pch-2(tm1458)* | 4.19 | 0.81 | 27 |
| *pch-2(tm1458);smrc-1(ea8)* | 6.05^N.S.^ | 1.32 | 21 |
| *cep-1(lg12501)* | 5.29 | 0.43 | 21 |
| *cep-1(lg12501);smrc-1(ea8)* | 2.70^$$$,&&^ | 0.65 | 20 |

***P<0.0001 compared to N2. N.S., not significantly different from single mutant controls. ^$$$^P<0.0001 compared to *smrc-1*. ^&&^P<0.002 compared to *cep-1.*
