# Supplementary material for: A DNA repair protein and histone methyltransferase interact to promote genome stability in the Caenorhabditis elegans germ line
Source: PLoS Genet. 2019 Feb 22;15(2):e1007992. doi: 10.1371/journal.pgen.1007992 (PMC6402707; doi:10.1371/journal.pgen.1007992)
Supplement: S1 Text — (DOCX) [file pgen.1007992.s008.docx]

**S1 Text. Supplemental Materials and methods.**

**Nematode culture and genetics**

Standard culture conditions were used [2]. The following mutations, balancer chromosomes, and transgenes were used in this study (see www.wormbase.org). LGI: *cep-1(gk138)*, *dog-1(gk10)*, *ego-1(om84).* LGIII: *met-2(n4256)*, *3xflag::met-2 [3]* (strain EL634), *set-25(tm5021)*, *smrc-1(om136)*, *smrc-1(om138)*, *smrc-1(ea8)*, and *smrc-1(ea46)* (this study), *3xflag::smrc-1* (strains EL610) and *3xmyc::smrc-1* (strain EL663; also contains *3xflag::met-2*) (this study), *hT2 [bli-4(e937) let-?(q782) qIs48] (*called *hT2 gfp*, below)*, qC1 [dpy-19(e1259) glp-1(q339) nIs189[myo-2::gfp]]*, *qC1 [dpy-19(e1259) glp-1(q339) qIs26[lag-2::gfp rol-6(su1006)]]*. LGIV: *csr-1(tm892)*, *him-8(e1489)*; *spo-11(me44)*, *spo-11(ok79)*, *nT1*, *ltIs37[pie-1p::mCherry::his-58+unc-119(+)]*. LGV: *bcIs39 [plim-7::ced-1::GFP+lin15(+)].* LGX: *unc-58(e665).* To generate a *smrc-1 met-2* double mutant, *smrc-1(om138)* was generated by CRISPR-mediated editing in the *met-2(n4256)/qC1* background. Homozygous *smrc-1(om138) met-2(n4256)* was outcrossed twice and rebalanced (strain EL632). The *smrc-1(om136) set-25(tm5021)* double mutant, *met-2(n4256) set-25(tm5021)* double mutant, and *smrc-1(om138) met-2(n4256) set-25(tm5021)* triple mutant strains (EL638, EL639, and EL679, respectively) were generated by conventional recombination, and mutations were confirmed by DNA amplification; double and triple mutant chromosomes were maintained over the *hT2 gfp* balancer described above. The *smrc-1(om138) 3xflag::met-2* chromosome was made by using CRISPR editing to generate the *om138* frameshift mutation on the *3xflag::met-2* chromosome (strain EL658). A *mex-5p::met-2::gfp::3xflag::met-2 3’UTR* transgene, *omIs1*, was generated by mos1-mediated single copy insertion (mosSCI) as described below.

**CRISPR**

The *dpy-10* co-CRISPR strategy was adapted to facilitate selection of transformants [4, 5]. Unique CRISPR guides near the start codons of *smrc-1* and *met-2* were selected under the guidance of the CRISPR design website <http://crispr.mit.edu>. To generate the *om136* nonsense and *om138* frameshift mutations, primers matching the guide sequences were incorporated into the pDD162 vector through overlapping PCR. Repair templates to introduce mutations through homologous recombination were purchased from Invitrogen. To generate epitope-tagged transgenes, repair templates containing 33bp flanking sequences and the appropriate tag sequence (*flag* or *myc*) were amplified by high fidelity PCR and purified. Injection mixes contained the following DNAs: 50 ng/ul Cas9 nuclease plasmid; 25 ng/μl *dpy-10(cn64)* repair template (100 nucleotide oligo); 25 ng/μl each guide RNA (gRNA) plasmid (*dpy-10* and the gene of interest); 50ng/ul repair template for gene of interest (PCR product). Roller progeny of injected adults were picked to single plates and allowed to lay eggs before being assayed by single worm PCR. The *smrc-1(ea8)* frameshift allele was generated through non-homologous end joining (no repair template was introduced); an sgRNA downstream of the *smrc-1* start codon was cloned into pRB1017. A DNA mixture containing sgRNA vectors for *smrc-1* and *dpy-10*, a DNA repair oligonucleotide to create *dpy-10(cn64),* and pDD162 Peft-3::Cas9 was injected into the gonads of N2 day 1 adult hermaphrodites. The *smrc-1(ea46)* deletion allele was generated by using synthetic crRNA (2 nmol IDT) targeting sites near the 5’ start codon and 3’ stop codon. A mixture of crRNA, tracrRNA, purified Cas9 enzyme, and a single-stranded oligonucleotide repair template was injected.

**mosSCI**

The *mex-5* promoter was amplified from genomic DNA using the following primers: 5’- cctaggtcacaacggcaaaatatcag-3’ and 5’-cattctctgtctgaaacattcaattg-3’ [6]; an AvrII restriction enzyme site was introduced for cloning into pCFJ151 [7]. The *met-2* coding sequences were amplified from genomic DNA. Coding sequences for GFP and 3×FLAG were amplified from plasmid provided by Dr. David Greenstein. The epitope tag module was inserted at last codon before the stop codon using overlapping PCR. The *mex-5p::met-2::gfp::3xflag::met-2 3’UTR* was inserted into pCFJ151 vector. The final construct was injected at 50ng/μl along with pCFJ90 (2.5ng/μl), pGH8 (10ng/μl), pCFJ104 (5ng/μl), pCFJ601 (30ng/μl) and pMA122 (10ng/μl) into EG6699 bearing the ttTi5605 Mos1 transposon [7].

**DAPI staining and clutch/brood size assays**

DAPI-staining was performed as described [8]. The numbers of embryos (clutch size), viable progeny (brood size), and fertile/infertile adults produced were determine according to standard methods, as follows. L4 hermaphrodites were singled onto freshly seeded plates; as egg-laying adults, they were transferred to new plates every 24 hours. The number of embryos on each plate was recorded immediately after the hermaphrodite was removed. After 2 days (at 25°C) or 3 days (at 20°C) the numbers of fertile adult hermaphrodites, sterile adult hermaphrodites, and adult males were recorded for each plate.

**Protein blots**

Nematodes were grown to appropriate stage on nematode growth medium (NGM) plates and

then harvested by rinsing the plates with M9 buffer. Worm pellets were collected by centrifuging at 13,200 rpm for 1 min; the supernatant was aspirated, a volume of sample buffer equal to the pellet volume was added to the tube, and material was resuspended. Material was boiled at 95°C for 10 mins with periodic vortexing. Extracts were resolved on SDS-PAGE (7.5% -15% gradient) and transferred to nitrocellulose membrane. Membranes were incubated with primary antibody (anti-FLAG, Sigma, 1:500; anti-MET-2, this study, 1:500) overnight at 4°C, washed, and incubated with horseradish peroxide-conjugated secondary antibody (1:5000). Signal was visualized with ECL substrate (Pierce).

**Immunohistochemistry**

Indirect immunolabeling of dissected gonads was carried out as previously described [9-11]. In brief, adults (24 hour post-L4) were dissected in PBS + 0.2mM levamisole in deep-well slides. Tissue was fixed with 3% PFA for 5 mins. For FLAG- and MYC-tagged protein detection, and for RNA:DNA hybrid detection, tissue was post-fixed in 100% -20°C methanol for 1 min. For H3S10phos detection, the methanol post-fix was 1 hr. Primary antibodies were mouse anti-FLAG (Sigma, 1:200), mouse anti-H3K9me2 (Abcam 1220, 1:200, rabbit anti-MYC (Invitrogen, 1:200), mouse monoclonal anti-H3S10phos (Millipore clone 3H10, 1:200), and mouse monoclonal anti-RNA:DNA hybrid (Millipore clone S9.6, 1:200). Secondary antibodies were Alexafluor 488-conjugated goat anti-mouse (1:200) and Alexafluor 568-conjugated donkey anti-rabbit (1:200).

The freeze-crack procedure used to co-label 3XFLAG::MET-2 and 3XMYC::SMRC-1 was adapted from Phillips *et al.,* 2009 [12]. In brief, 24-hr post-L4 adults were washed twice in M9, transferred to 30 µl of Egg buffer/0.1% Tween-20/0.2 mM levamisole on a coverslip, and dissected. 30 µl of 5% PFA in Egg Buffer was immediately added as fixative; solution was pipetted up and down to mix thoroughly. After 5 min, 45 µl of solution was removed, and a Superfrost^®^ slide was added on top of the remaining solution. The tissue sandwiched between coverslip and Superfrost^®^ slide was inverted and gently tapped to rupture cells. Tissue was placed on the surface of a pre-chilled metal block on dry ice for > 20 minutes; coverslip was popped off, and pre-chilled 100% methanol was added to the tissue for 1 min. Tissue was washed three times with PBST, and blocked in PBST/30% goat serum for 30 minutes at room temperature. Blocking reagent was removed, primary antibodies diluted in PBST/GS were added, and sample was incubated overnight at 15°C. Primary antibody solution was removed; tissue was washed 3x 15 min in PBST. Secondary antibodies diluted in PBST/GS were added and tissue was incubated for 2 hr at room temperature in the dark. Tissue was washed 1x in PBS, incubated in DAPI/PBS for 15 min, and washed a final time in PBS. All solution was removed, ~4ul Vectashield^®^ and a coverslip were added, and slides were stored at 4°C.

We note that H3K9me2 abundance appears normal in strains with endogenous *met-2* tagged at the N-terminus via CRISPR (Figure S1C). Therefore, the N-terminal tag does not appear to decrease MET-2 activity. In contrast, H3K9me2 signal is moderately reduced in mosSCI lines carrying a C-terminal tag on the *met-2* transgene; this is the case even when the endogenous *met-2* gene is deleted and despite the fact that the *met-2(n4256)* reduced brood size phenotype was fully rescued in these lines (S1E Fig).

**Quantification of immunolabeling images**

To quantify immunolabelling intensity of mitotic 3XFLAG::MET-2 and 3XFLAG:SMRC-1, images containing the 3xFLAG signal as well as DAPI, mCherry::HIS-58, or pan-H3 labeling were captured at fixed exposure times in different channels and imported separately into ImageJ. The three channels were synchronized using the tool manager; circles were drawn at the periphery of each nucleus in the DAPI channel and then copied into the other channels. Mean intensities were measured in each channel, and quantification was calculated by normalizing the 3xFLAG signal to DAPI, mCherry::HIS-58, or pan-H3 signal, accordingly.

H3K9me2 labeling intensity was calculated using a variation on the corrected total cell fluorescence (CTCF) method previously described [13, 14]. To calculate the corrected total nuclear fluorescence (CTNF), H3K9me2 and DAPI labeling images were imported into ImageJ (NIH). A line was drawn around the periphery of each nucleus, and measurements of integrated density, area, mean, min and max grey values were recorded. Background fluorescence was measured as follows. For H3K9me2 labeling in meiotic nuclei as shown in Fig 9, a 10 x 10 pixel area in the cytoplasm was selected as background. For H3K9me2 labeling in the distal germ line, a 10 x 10 pixel area adjacent to tissue section was selected as background. CTNF was calculated as [integrated density – (area X mean grey value of background)].

To quantify S9.6 labeling, we counted the total number of nuclei and the number of S9.6-positive nuclei within each of three germline regions (proliferative, leptotene-zygotene, pachytene) for a set of germ lines. We averaged the values and calculated the S9.6 index for each region by dividing the average number of S9.6-positive nuclei by the average total number of nuclei.

**Hydroxyurea assay**

Assays were carried out as previously described [15]. In brief, Hydroxyurea (HU) was dissolved in M9 to a stock concentration of 2M. HU stock solution was further diluted with M9 (final volume of 250 µl) and spread on to 6 cm plates containing 10ml of NGM media, final concentrations of 2.5mM, 5mM, 10mM and 25mM HU were prepared. HU solution was left to soak into NGM media overnight, and plates were used within 24 hours. HU plates were seeded with OP50 and dried for 2 hours under a fume hood. For the L1 assay, larvae were picked from NGM plates and transferred to HU-soaked NGM plates; animals were incubated at 25 °C for 16 hours, and then transferred to freshly seeded NGM plates. After 48 hours, viable adults were scored and survival rates were calculated. To generate animals for immunolabeling, L4 larvae were placed onto HU-soaked NGM plates at 22°C for 16 hr, dissected (as adults), fixed, and immunolabeled as appropriate to visualize SMRC-1, MET-2, or H3S10phos. Tissue was counter-stained with DAPI in the penultimate wash. The mitotic index for each germ line was calculated as the number of H3S10phos-positive nuclei distal to meiotic entry/ total number of nuclei distal to meiotic entry.

**Immunoprecipitation and mass spectrometry**

MET-2 IP was performed with nuclear extract was prepared from *him-8(e1489)* adults. 500-750 µl of pelleted worms were resuspended in 1 ml HB buffer (150 mM Hepes pH 7.6, 10 mM KCl, 5 mM MgCl_2_, 12% sucrose, 0.1 mM EDTA) and the volume split into two tubes. 100 µl glass beads (710-1180 microns) was added to each tube, and tissue was disrupted using a FastPrep 120 for two 15 sec pulses on setting 5.5s, with 10 min on ice in between pulses. Beads were pelleted at 800 rpm for 2 min in a microfuge. Supernatants were pooled into a clean tube, NP40 added to a final concentration of 0.2%, and tube stored on ice for 30 min. Tube was spun at 8000 rpm for 2 min; pellet was washed by pipetting up and down to disperse in 1 ml of HB solution and spun again at 8000 rpm for 2 min. Pellet was resuspended in HS solution (300 mM NaCl, 20 mM Tris pH 7.6, 25% glycerol, 1.5 mM MgCl_2_, 0.2 mM EDTA), and sufficient volume of 900 mM NaCl was added to adjust final concentration to 300 mM NaCl. Protease inhibitor cocktail (Roche) was added and tube placed on ice for 30 min. Extract was spun at 10000 rpm for 30 min at 4°C to pellet debris. Protein concentration was determined by Bradford assay. To IP MET-2, 3-5 µg of anti-MET-2 antibody was incubated with ~300-500 ng of protein overnight at 4°C with rotation. After removing an aliquot to use as input, an appropriate volume of precleared Protein A/G beads was added and incubated for > 2 hr at 4°C with rotation. Beads were pelleted, washed twice RT with FA buffer (1 mM EDTA, 50 mM HEPES-KOH, 140 mM NaCl, 0.1% sodium deoxycholate, 1% Triton X-100) and once with FA-500 (FA with 500 mM NaCl), and protein was eluted at RT for 10 min with 2X SDS loading buffer. Beads were pelleted at 2000 rpm for 1-2 min. Supernatant was removed to a new tube, beta-mercaptoethanol was added, tube was place at 95°C for 10 min, vortexed, and spun to pellet debris. Supernatant was loaded onto a protein gel. Using size standards (Bio-rad, Precision Plus protein standards) as a guide, the gel lane was cut into 4 pieces for tandem mass spec analysis of proteins (Keck Facility, Yale).

3xFLAG::SMRC-1 IP and immunoblot analysis were performed as follows. Anti-FLAG antibodies were coupled with protein G Dynabeads (Invitrogen) to reach a final concentration of 5µg antibody/mg beads following the Invitrogen protocol. Synchronized worms were harvested from ten 60 mm NGM plates and collected by centrifuging at 13,200 rpm for 1min. Supernatants were aspirated, worm pellets were snap frozen in liquid nitrogen and stored in -80°C freezer until use. Worm pellets were thawed on ice and an equal volume of H-100 buffer containing NP40 (50 mM Hepes, pH 7.5, 100 mM KCl, 1mM MgCl2, 1 mM EDTA, 10% glycerol); proteinase inhibitor cocktail (Roche) was added to the tube. Extracts were prepared using a FastPrep as described above. 3-5 mg of protein extract was incubated with 1 mg of antibody-coupled Dynabeads overnight at 4°C with rotation. Beads were washed 2X 5 min with 1ml of H-100 buffer. Proteins were eluted with 20ul of sample buffer at 55°C for 10 min.

**Recombination assays**

Recombination between visible markers was measured by standard methods [16]. We generated *dpy unc /++; smrc-1(+)* and *dpy unc/++;* *smrc-1(om136)/qC1* strains carrying either *unc-11 dpy-5* or *dpy-5 unc-13.* We scored full broods from a set of *dpy unc /++; smrc-1(+)* and *dpy unc/++;* *smrc-1(om136)* hermaphrodites for Dpy Unc, Dpy non-Unc, and Unc non-Dpy, and non-Dpy non-Unc phenotypes. The measureable recombination rate is Dpy non-Unc + Unc non-Dpy/total. This value represents approximately half of the total recombinants, as calculated according to Brenner [91]. S4B Fig lists the measured value. The *smrc-1* mutation was maintained over the *qC1* balancer chromosome until the generation to be assayed. Whole chromosome recombination was assayed in oocytes as described [17]. A *smrc-1* deletion allele was induced in the polymorphic CB4256 (“Hawaiian”) strain using CRISPR. Heterozygous *smrc-1*^CB^/*smrc-1*^N2^ hermaphrodites were mated with N2 wildtype males carrying an X-linked *gfp* marker; F1 hermaphrodite cross-progeny were identified based on GFP expression, picked to single plates, and allowed to produce offspring. Restriction fragment length polymorphisms (RFLPs) were evaluated in at six positions along chromosome I.

***unc-58* suppression and *dog-1* enhancement**

We assayed for suppression/reversion of the *unc-58(e665)* phenotype as described [18] in *unc-58* control and *smrc-1(ea8);unc-58* mutants raised at 20°C. We evaluated enhancement of the *dog-1* phenotype by assaying for accumulation of insertions and deletions within the GC-rich exon 5 of *vab-1* as described [19] in *dog-1(gk10)* controls, *smrc-1(ea8)* single mutants, and *dog-1(ea8);smrc-1(ea8)* double mutants grown at 20° and 25°C. We also tested *dog-1(gk10);met-2(n4256)* double mutants and *dog-1(gk10);smrc-1(om138) met-2(n4256)* triple mutants at 25°C. Nested DNA amplification was performed with oligonucleotides flanking *vab-1* exon 5. Before performing the assay, strains were outcrossed to eliminate any *vab-1* deletions that might have accumulated and were screened to confirm the absence of deletion.

**Transgenerational broods and sterility**

Six lines of balanced *smrc-1(ea8)/qC1* were maintained at 25°C for three generations. They were expanded to 16 unbalanced founders on 12-well NGM plates seeded with OP50. To avoid bias towards healthy animals, the first L4 offspring on each plate was passaged to start the next generation. Two days after passaging, the total number of progeny in the previous generation was estimated using the following categories: 0, <20, 20-40, 40-80, 80-100, and >100. If a worm failed to produce progeny, became eviscerated, or otherwise died before any offspring were produced, the line was rescued with a healthy sibling. Sterile worms were scored as zero progeny and eviscerated/dead worms were not scored.

**Supplemental references**

1. Li Y, Maine EM. The balance of poly(U) polymerase activity ensures germline identity, survival and development in *Caenorhabditis elegans*. Development. 2018;145(19):dev165944.

2. Epstein HF, Shakes DC. Preface. In: Epstein HF, Shakes DC, editors. Methods in Cell Biology. 48: Academic Press; 1995.

3. Mutlu B, Chen H-M, Moresco JJ, Orelo BD, Yang B, Gaspar JM, et al. Regulated nuclear accumulation of a histone methyltransferase times the onset of heterochromatin formation in *C. elegans* embryos. 2018;4(8):eaat6224.

4. Paix A, Wang Y, Smith HE, Lee CY, Calidas D, Lu T, et al. Scalable and versatile genome editing using linear DNAs with microhomology to Cas9 Sites in Caenorhabditis elegans. Genetics. 2014;198(4):1347-56.

5. Arribere JA, Bell RT, Fu BX, Artiles KL, Hartman PS, Fire AZ. Efficient marker-free recovery of custom genetic modifications with CRISPR/Cas9 in Caenorhabditis elegans. Genetics. 2014;198(3):837-46.

6. Merritt C, Rasoloson D, Ko D, Seydoux G. 3' UTRs are the primary regulators of gene expression in the C. elegans germline. Curr Biol. 2008;18(19):1476-82.

7. Frokjaer-Jensen C, Davis MW, Sarov M, Taylor J, Flibotte S, LaBella M, et al. Random and targeted transgene insertion in Caenorhabditis elegans using a modified Mos1 transposon. Nat Methods. 2014;11(5):529-34.

8. Qiao L, Lissemore JL, Shu P, Smardon A, Gelber MB, Maine EM. Enhancers of glp-1, a gene required for cell-signaling in Caenorhabditis elegans, define a set of genes required for germline development. Genetics. 1995;141(2):551-69.

9. She X, Xu X, Fedotov A, Kelly WG, Maine EM. Regulation of heterochromatin assembly on unpaired chromosomes during Caenorhabditis elegans meiosis by components of a small RNA-mediated pathway. PLoS Genet. 2009;5(8):e1000624.

10. Maine EM, Hauth J, Ratliff T, Vought VE, She X, Kelly WG. EGO-1, a putative RNA-dependent RNA polymerase, is required for heterochromatin assembly on unpaired dna during C. elegans meiosis. Current biology : CB. 2005;15(21):1972-8.

11. Guo Y, Yang B, Li Y, Xu X, Maine EM. Enrichment of H3K9me2 on Unsynapsed Chromatin in *Caenorhabditis elegans* Does Not Target *de Novo* Sites. G3: Genes|Genomes|Genetics. 2015;5(9):1865.

12. Phillips CM, McDonald KL, Dernburg AF. Cytological analysis of meiosis in Caenorhabditis elegans. Methods Mol Biol. 2009;558:171-95.

13. McCloy RA, Rogers S, Caldon CE, Lorca T, Castro A, Burgess A. Partial inhibition of Cdk1 in G2 phase overrides the SAC and decouples mitotic events. Cell Cycle. 2014;13(9):1400-12.

14. Ow MC, Borziak K, Nichitean AM, Dorus S, Hall SE. Early experiences mediate distinct adult gene expression and reproductive programs in Caenorhabditis elegans. PLoS genetics. 2018;14(2):e1007219-e.

15. Craig AL, Moser SC, Bailly AP, Gartner A. Methods for Studying the DNA Damage Response in the Caenorhabdatis elegans Germ Line. In: Rothman JH, Singson A, editors. Methods in Cell Biology. 107: Academic Press; 2012. p. 321-52.

16. Kelly KO, Dernburg AF, Stanfield GM, Villeneuve AM. Caenorhabditis elegans msh-5 is required for both normal and radiation-induced meiotic crossing over but not for completion of meiosis. Genetics. 2000;156(2):617-30.

17. Wagner CR, Kuervers L, Baillie DL, Yanowitz JL. xnd-1 regulates the global recombination landscape in Caenorhabditis elegans. Nature. 2010;467(7317):839-43.

18. Harris J, Lowden M, Clejan I, Tzoneva M, Thomas JH, Hodgkin J, et al. Mutator phenotype of Caenorhabditis elegans DNA damage checkpoint mutants. Genetics. 2006;174(2):601-16.

19. Youds JL, O'Neil NJ, Rose AM. Homologous recombination is required for genome stability in the absence of DOG-1 in Caenorhabditis elegans. Genetics. 2006;173(2):697-708.
